# Dose titration of osmotic release oral system methylphenidate in children and adolescents with attention-deficit hyperactivity disorder: a retrospective cohort study

**DOI:** 10.1186/s12887-023-03850-4

**Published:** 2023-01-23

**Authors:** Youran Xu, Hsingwen Chung, Meng Shu, Yanfang Liu, Yongjing Zhang, Hong Qiu

**Affiliations:** 1 Global Epidemiology, Office of Chief Medical Officer, Johnson & Johnson, Beijing, China; 2 Global Epidemiology, Office of Chief Medical Officer, Johnson & Johnson, Titusville, NJ USA; 3 Global Epidemiology, Office of Chief Medical Officer, Johnson & Johnson, Shanghai, China; 4 Global Epidemiology, Office of Chief Medical Officer, Johnson & Johnson, Singapore, Singapore

**Keywords:** Osmotic release oral system methylphenidate (OROS-MPH), Attention-deficit hyperactivity disorder (ADHD), Dose titration, Real-world evidence, Children and adolescents

## Abstract

**Background:**

Osmotic release oral system methylphenidate (OROS-MPH) is one of the most commonly used medication for attention-deficit hyperactivity disorder (ADHD), however, real-world knowledge on OROS-MPH dose titration has been limited. This study aims to summarize and visualise the OROS-MPH titration patterns in children and adolescents with ADHD in the United States (US) and Japan.

**Methods:**

This retrospective cohort study used the US IBM® MarketScan® Commercial Claims and Encounters database from 2000 to 2019 and the Japan Medical Data Centre database from 2008 to 2019. New OROS-MPH users with ADHD were identified and split into child (6 to < 13 years) and adolescent (13 to < 18 years) groups according to age at OROS-MPH initiation/reinitiation. Patient characteristics and OROS-MPH treatment patterns were described. OROS-MPH dose titration pathways were visualised by Sankey diagrams.

**Results:**

We included 98,973 children and 62,002 adolescents in the US cohort, and 4595 children and 1508 adolescents in the Japanese cohort. In Japanese cohort, 91.9% of children and 77.9% of adolescents initiated OROS-MPH at the lowest dose (18 mg/day), whereas US patients had a broader distribution of initial doses (e.g., 18–54 mg/day). The US patients had higher daily dose of OROS-MPH than Japanese patients. Overall, a minority (< 40%) of the OROS-MPH users underwent dose titration, and different titration patterns were observed between the US and Japanese patients.

**Conclusions:**

Different treatment and titration patterns of OROS-MPH were observed in the two countries. Additional real-world studies about clinical reasoning underlying dose selection are needed to support clinical decision-making.

**Supplementary Information:**

The online version contains supplementary material available at 10.1186/s12887-023-03850-4.

## Background

Attention-deficit hyperactivity disorder (ADHD) is a common psychiatric disorder among children, affecting around 5–7% of the population younger than 18 years of age worldwide [1–6]. Although symptom onset is typically during early to mid-childhood, the disorder may persist into adolescence or adulthood [2, 7–10]. Even though the estimated prevalence of ADHD varies little between North America and Asia [2, 4, 11–13], the prevalence of ADHD medication use in Asia has been reported to be much lower than in North America (2010: 4.5% in North America vs. 1.0% in Asia) [14]. Medications available for the treatment of ADHD include psychostimulants and non-psychostimulants. For school-age populations with ADHD, psychostimulants are recommended for first-line treatment and the United Kingdom National Institute for Health and Care Excellence (NICE) ranks methylphenidate (MPH) over other psychostimulants [15–18]. A multi-country population-based observational study covering 150 million individuals reported that MPH was the most widely used ADHD medication in most countries, including the United States (US) (US Medicaid population) and Japan [14]. Non-psychostimulants are used for second-line treatment if psychostimulants prove inadequate or intolerable [2].

MPH comes in immediate release (IR) and extended release (ER) formulations, and ER MPH has become increasingly dominant in recent years [19, 20]. Osmotic release oral system methylphenidate (OROS-MPH, Concerta®; Janssen Pharmaceuticals, Inc) is an ER-MPH tablet that should be swallowed whole. Due to its once-daily dosing and equivalent effectiveness to other MPH formulations [21–23], OROS-MPH has been quickly and widely accepted as the preferred treatment for ADHD in Western countries [21, 24, 25]. In addition, OROS-MPH was the only approved psychostimulant for ADHD indication in Japan until 2019 [26, 27].

MPH should be initiated at a low dose, especially in children due to their slower drug metabolism and variable drug responses [18]. Upward dose titration is recommended to achieve a target dose that optimises effectiveness and minimises side-effects [18, 28]. According to OROS-MPH drug labels in the US and Japan [29, 30], patients with ADHD younger than 18 years old are recommended to initiate treatment at 18 mg/day, and increase the daily dose in 9 or 18 mg increments when titration is needed. These recommendations derive primarily from clinical trials [31, 32], however, evidence reflecting the titration strategies for OROS-MPH in real-world settings is limited. To fill this gap, we conducted a retrospective cohort study in children and adolescents with ADHD using two claims databases from the US and Japan, where OROS-MPH has been used for long times and extensive drug utilization experience has been accumulated. We aimed specially to describe OROS-MPH consumption and titration patterns in these two countries and to provide real-world evidence to support clinical decision making.

## Methods

### Study drug

OROS-MPH drug codes were defined as ‘methylphenidate’ as the generic name and ‘Concerta’ as the brand name in the study databases. In the US, OROS-MPH was approved for child and adolescent ADHD indications in 2000 and 2004 respectively, and four tablet strengths are available (18, 27, 36 and 54 mg). OROS-MPH was approved in Japan in late 2007 for both child and adolescent populations and three tablet strengths are available (18, 27 and 36 mg).

### Data sources

This retrospective cohort study utilised national health insurance claims data from the IBM® MarketScan® Commercial Claims and Encounters (CCAE) database in the US for the period 2000–2019, and the Japan Medical Data Centre (JMDC) database for the period 2008–2019. To obtain sufficient observation period, retrospective data extraction from CCAE began in the year of OROS-MPH approval in the US, i.e., year 2000. In Japan, due to the lack of data of 2007, when OROS-MPH was approved, data collection began on January 1, 2008. The CCAE database covers approximately 157 million people and these data include health insurance claims across the continuum of care (e.g., inpatient, outpatient and outpatient pharmacy) as well as enrolment data from large employers and health plans across the US who provide private healthcare coverage for employees, their spouses, and dependents. The JMDC is a similar payer-based insurance claims database covering more than 13 million non-government employees and their families in Japan. The JMDC captures monthly claims issued by clinics, hospitals and community pharmacies. Both the CCAE and JMDC are high-quality commercial claims databases including representative data on children and adolescents from the two countries.

### Study populations

We first identified patients with a diagnosis of ADHD (ICD-10-CM: F90.x or ICD-9-CM: 314. xx) in the CCAE from 01 January 2000 to 31 December 2019, and in the JMDC from 01 January 2008 to 31 December 2019, reflecting the OROS-MPH launch date in each country. Patients with ADHD who had at least one OROS-MPH prescription on the same day or after the first ADHD diagnosis were identified. Patients were included if they used OROS-MPH when aged between 6 and < 18 years of age, and if they had at least 6 months of continuous enrolment in the database prior to OROS-MPH initiation. Patients who initiated OROS-MPH between 6 and < 13 years of age were grouped into the child group, and patients who initiated OROS-MPH between 13 and < 18 years of age were grouped into the adolescent group. Patients in the child group who reinitiated OROS-MPH (i.e., recommenced treatment following a ≥ 6-month OROS-free period) between 13 and < 18 years of age were also included into the adolescent group. Thus, some patients were categorised in both the child and adolescent groups. Index date referred to the date when OROS-MPH was first used during this age group. Patients who were enrolled for < 1 year following the index date were excluded. Patients who used OROS-MPH earlier than its launch date (i.e., off-label users) were also excluded.

### Data collection

Data were collected relating to the index episode, which was from the index date until the first drug discontinuation of OROS-MPH. Discontinuation was defined as a gap of 6 months after the end of the previous prescription period, or death, end of enrolment, or end of the study (31 December 2019), whichever occurred first. Index episodes could persist into the next life stage (e.g., from childhood to adolescence or from adolescence to adulthood), which would not interrupt data collection. Demographic variables (gender and age) and baseline clinical characteristics including comorbid psychiatric disorders (major depressive, anxiety, learning disorders, oppositional behaviour, and substance-related disorders) and ADHD medication history (psychostimulants and non-psychostimulants) were extracted from insurance claims within 6 months prior to the index dates. OROS-MPH prescription information during the index episodes was collected, including diagnosis, drug name, prescription date, dose of each tablet, daily dose, and overall quantity delivered.

### Statistical analysis

Data analyses were conducted in child and adolescent groups separately for the US and Japanese cohorts. Baseline demographic and clinical characteristics were described. Age was reported as a continuous variable (median, inter-quartile range [IQR]). Gender, comorbid psychiatric disorders and ADHD medication history at baseline were reported as categorical variables.

The OROS-MPH daily dose and overall consumption during the index episode were described. The initial regimen was measured categorically as monotherapy vs. combination (with other ADHD medications). OROS-MPH initial, maintenance, maximum, and average daily doses were summarised as both continuous and categorical variables. The maintenance dose was the daily dose level used continuously for the longest time and at least 60 days. The proportion of patients who had maintenance dose was calculated. Because OROS-MPH tablets need to be swallowed whole, the daily dose should be equal to the dose of one single tablet or the sum of multiple tablets. As a result, initial, maintenance, and maximum daily dose were categorised as 18, 27, 36, 45, 54, and > 54 mg/day, and average daily dose was categorised as < 19, 19–27, 28–36, 37–45, 46–54 and > 54 mg/day. Duration of the index episode was reported both continuously and categorically (< 6 months, 6–12 months, and > 12 months), and the number of OROS-MPH prescriptions were reported categorically (1, 2–3, 4–9, 10–20, and > 20).

Numbers of dose titration for each patient were reported categorically (0, 1, 2 or ≥ 3), and for patients in each category the durations of index episodes were described. OROS-MPH titration pathways were visualised by Sankey diagrams for the patients with at least one dose titration. The graphs were constructed as follows: the first column of bars represents the initial dose level, and patient movement to the second column of bars represents titration to the second dose level, and so on.

Sensitivity analyses were conducted among the US patients who had never been exposed to any generic MPH within 6 months prior to OROS-MPH initiation (i.e., MPH-naive patients) to identify if previous experience with MPH influenced OROS-MPH initial dose and titration pattern.

## Results

### Baseline characteristics

**Fig. 1:**
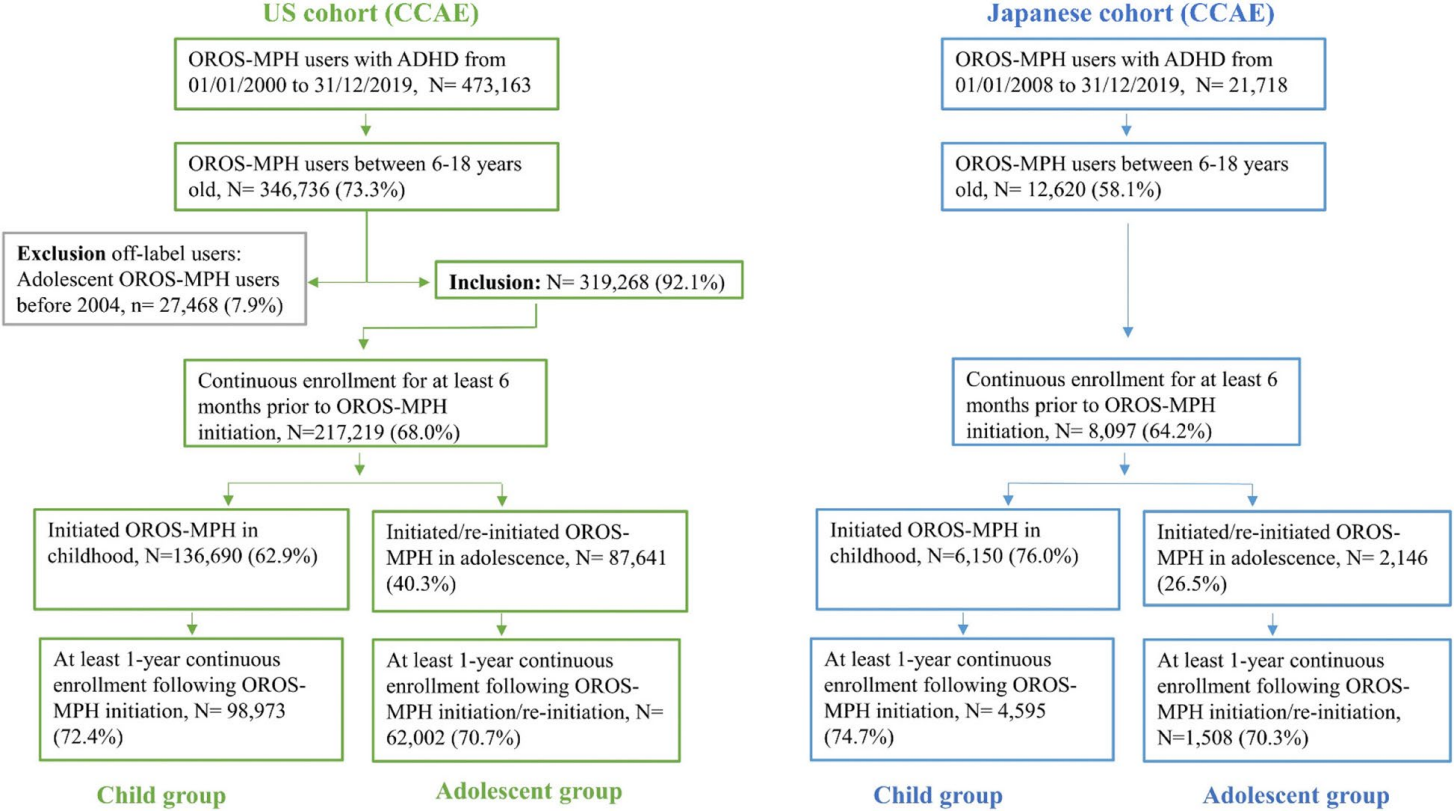
Flowchart of study cohort selection in the US and Japanese cohorts. Childhood 6 to < 13 years of age; adolescence: 13 to < 18 years of age

After application of inclusion/exclusion criteria, 98,973 child patients and 62,002 adolescent patients were included in the analysis for the US cohort. Among the US adolescents, 11.5% (*n* = 7112) had been exposed to OROS-MPH during both of childhood and adolescence. In the Japanese cohort, 4595 child patients and 1508 adolescent patients were included in the analyses. Among Japanese adolescents, 8.5% (*n* = 128) had also been exposed to OROS-MPH during their childhood (Fig. 1).

Age and gender distributions were similar in the US and Japanese cohorts (Table 1). In both countries the median age at OROS-MPH initiation was 9 years for children and 14–15 years for adolescents, and 67.8–85.4% of patients were male. Baseline comorbid psychiatric disorders were more frequently observed in the adolescent group than the child group in both countries (16.8% vs. 10.9% for the US cohort and 23.4% vs. 9.2% for the Japanese cohort). The most common comorbid psychiatric disorder was anxiety in US children, learning disorder in Japanese children and major depressive disorder in adolescents in both countries. In the US cohort, 46.0% of children and 39.4% of adolescents had used other ADHD medications prior to OROS-MPH, versus 17.1 and 21.2%, respectively in Japan. In the US cohort, 40.7% of children and 34.8% of adolescents had previously used psychostimulants (mainly generic MPH) and 11.9 and 9.6%, respectively had used non-psychostimulants. By contrast, in the Japanese cohort, < 1% of children and adolescents had previously used psychostimulants, and 16.7 and 21.0%, respectively had used non-psychostimulants (usually atomoxetine), before OROS-MPH.

**Table 1:** Demographic and clinical characteristics of OROS-MPH users with ADHD in the US and Japan, by age groups

	United States		Japan	
	Child group (***n*** = 98,973)	Adolescent group (***n*** = 62,002)	Child group (***n*** = 4595)	Adolescent group (***n*** = 1508)
**Median age at index date, years (IQR)**	9 (8–11)	15 (14–16)	9 (8–11)	14 (14–16)
**Sex, n (%)**				
Male	71,559 (72.3)	42,020 (67.8)	3923 (85.4)	1102 (73.1)
Female	27,414 (27.7)	19,982 (32.2)	672 (14.6)	406 (26.9)
**Comorbid psychiatric disorders**^**a**^**, n (%)**				
Overall	10,819 (10.9)	10,439 (16.8)	424 (9.2)	353 (23.4)
Major depressive	2466 (2.5)	5236 (8.4)	96 (2.1)	220 (14.6)
Anxiety	4844 (4.9)	4647 (7.5)	127 (2.8)	106 (7.0)
Learning disorder	1989 (2.0)	809 (1.3)	181 (3.9)	66 (4.4)
Oppositional behavior	2853 (2.9)	1815 (2.9)	30 (0.7)	10 (0.7)
Substance related disorders	32 (0.03)	305 (0.5)	10 (0.2)	4 (0.3)
**Other ADHD medication users before OROS-MPH**^**b**^**, n (%)**				
Overall	45,558 (46.0)	24,398 (39.4)	786 (17.1)	320 (21.2)
Psychostimulants:				
Overall	40,248 (40.7)	21,591 (34.8)	23 (0.5)	5 (0.3)
Other generic MPH	23,566 (23.8)	12,915 (20.8)	24 (0.5)	5 (0.3)
Amphetamine	11,775 (11.9)	5385 (8.7)	0	0
Other psychostimulants^c^	10,660 (10.8)	5581 (9.0)	0	0
Non-psychostimulants:				
Overall	11,815 (11.9)	5922 (9.6)	766 (16.7)	317 (21.0)
Atomoxetine	5978 (6.0)	3040 (4.9)	637 (13.9)	282 (18.7)
Guanfacine	3320 (3.4)	1678 (2.7)	185 (4.0)	51 (3.4)
Clonidine	3426 (3.5)	1613 (2.6)	0	0
Other non-psychostimulants^d^	0	0	1 (0.02)	9 (0.6)

*ADHD* Attention deficit hyperactivity disorder, *OROS-MPH* Osmotic release oral system methylphenidate, *IQR* Inter-quartile range, Child group: 6 to < 13 years; adolescent group: 13 to < 18 years

^a^Comorbid psychiatric disorders within 6 months prior to index episode; patients might have more than one disorder

^b^all ADHD medications within 6 months prior to index episode; patients might have more than one medication

^c^lisdexamfetamine, dextroamphetamine, methamphetamine, and dexmethylphenidate; d: modafinil and pemoline

### OROS-MPH dose and consumption

Most patients started OROS-MPH as monotherapy (about 97% in the US cohort and 90–92% in the Japanese cohort) (Table 2). The median initial dose was 18 mg/day for the child group and 36 mg/day for the adolescent group in the US cohort, versus 18 mg/day for both age groups in the Japanese cohort. In the US cohort, the median maintenance dose was 36 mg/day for both groups, versus 18 mg/day among children and 27 mg/day among adolescents in Japanese cohort. Compared to the Japanese cohort, patients in the US cohort also had higher medians of maximum dose and average daily dose. The median duration of the index episode was 9.6 months in child group and 6.4 months in adolescent group in the US cohort, versus 14.7 and 9.1 months, respectively, in Japanese cohort. Children and adolescents in the US cohort received fewer OROS-MPH prescriptions than the equivalent age group in Japanese cohort.

**Table 2:** OROS-MPH dose and consumption of OROS-MPH users with ADHD in the US and Japanese cohort, by age groups

	United States		Japan	
	Child group (***n*** = 98,973)	Adolescent group (***n*** = 62,002)	Child group (***n*** = 4595)	Adolescent group (***n*** = 1508)
**Therapy on OROS-MPH index date, n (%)**				
Monotherapy	95,755 (96.7)	60,029 (96.8)	4235 (92.2)	1358 (90.1)
Combination therapy^a^	3218 (3.3)	1973 (3.2)	360 (7.8)	150 (9.9)
**Initial daily dose, mg/day**				
Median initial dose (IQR)	18 (18–36)	36 (18–45)	18 (18–18)	18 (18–18)
18, n (%)	51,985 (52.5)	18,030 (29.1)	4223 (91.9)	1174 (77.9)
27, n (%)	15,784 (15.9)	8577 (13.8)	79 (1.7)	84 (5.6)
36, n (%)	18,869 (19.1)	19,227 (31.0)	84 (1.8)	86 (5.7)
45, n (%)	1157 (1.2)	1006 (1.6)	167(3.6)	100 (6.6)
54, n (%)	8262 (8.3)	11,918 (19.2)	7 (0.2)	7 (0.5)
> 54, n (%)	2916 (2.9)	3244 (5.2)	35 (0.8)	57 (3.8)
**Maintenance daily dose**^**b**^**, mg/day**				
N (%)	75,201 (76.0)	41,937 (67.6)	3433 (74.7)	961 (63.7)
Median maintenance dose (IQR)	36 (18–36)	36 (27–54)	18 (18–27)	27 (18–27)
18, n (%)	19,193 (25.5)	5721 (13.6)	2053 (59.8)	404 (42.0)
27, n (%)	16,138 (21.5)	6132 (14.6)	1013 (29.5)	367 (38.2)
36, n (%)	24,397 (32.4)	17,150 (40.9)	221 (6.4)	107 (11.1)
45, n (%)	591 (0.8)	350 (0.8)	84 (2.4)	67 (7.0)
54, n (%)	12,646 (16.8)	11,900 (28.4)	47 (1.4)	13 (1.4)
> 54, n (%)	2236 (3.0)	684 (1.6)	15 (0.4)	3 (0.3)
**Maximum daily dose, mg/day**				
Median maximum dose (IQR)	36 (27–54)	36 (36–54)	27 (18–36)	27 (18–45)
18, n (%)	20,621 (20.8)	7522 (12.1)	2013 (43.8)	470 (31.2)
27, n (%)	17,548 (17.7)	7209 (11.6)	1278 (27.8)	403 (26.7)
36, n (%)	27,284 (27.6)	18,776 (30.3)	321 (7.0)	181 (12.0)
45, n (%)	1499 (1.5)	623 (1.0)	702 (15.3)	224 (14.9)
54, n (%)	24,319 (24.6)	20,377 (32.9)	70 (1.5)	55 (3.6)
> 54, n (%)	7702 (7.8)	7495 (12.1)	211 (4.6)	175 (11.6)
**Average daily dose, mg/day**				
Median average daily dose (IQR)	30 (21–38)	36 (27–48)	20 (18–26)	24 (18–32)
< 19, n (%)	21,152 (21.4)	7610 (12.3)	2146 (46.7)	497 (33.0)
19–27, n (%)	25,159 (25.4)	9897 (16.0)	1712 (37.3)	524 (34.7
28–36, n (%)	26,181 (26.5)	19,014 (30.7)	506 (11.0)	253 (16.8)
37–45, n (%)	12,011 (12.1)	8526 (13.8)	145 (3.2)	128 (8.5)
46–54, n (%)	10,994 (11.1)	12,965 (20.9)	47 (1.0)	58 (3.8)
> 54, n (%)	3476 (3.5)	3990 (6.4)	39 (0.8)	48 (3.2)
**Duration of the index episode, months**				
Median duration (IQR)	9.6 (2.3–21.4)	6.4 (1.8–15.9)	14.7 (2.3–29.8)	9.1 (1.6–20.6)
< 6 months, n (%)	39,638 (40.0)	30,073 (48.5)	1617 (35.2)	651 (43.2)
6–12 month, n (%)	14,335 (14.5)	10,398 (16.8)	348 (7.6)	168 (11.1)
> 12 months, n (%)	45,000 (45.5)	21,531 (34.7)	2630 (57.2)	689 (45.7)
**Number of prescriptions within this episode, n (%)**				
1	20,006 (20.2)	15,275 (24.6)	830 (18.1)	271 (18.0)
2–3	16,182 (16.3)	14,273 (23.0)	504 (11.0)	239 (15.8)
4–9	25,870 (26.1)	18,012 (29.1)	739 (16.1)	333 (22.1)
10–20	22,289 (22.5)	10,208 (16.5)	1103 (24.0)	344 (22.8)
> 20	14,626 (14.8)	4234 (6.8)	1419 (30.9)	321 (21.3)

*ADHD* Attention deficit hyperactivity disorder, *OROS-MPH* Osmotic release oral system methylphenidate, *IQR* Inter-quartile range, Child group: 6 to < 13 years; adolescent group: 13 to < 18 years

^a^combination therapy was defined as prescribing other ADHD medication(s) along with OROS-MPH

^b^a daily dose level that was continuously used for the longest time and for at least 60 days

### OROS-MPH titration

In this study, 69.4% of US children, 81.7% of US adolescents, 64.3% of Japanese children and 61.7% of Japanese adolescents didn’t underwent dose titration and remained at the initial dose for the entire index episode (Table 3). Among these patients, approximately 30% (20,006/68,736 of US children, 15,275/50,637 of US adolescents, 830/2955 of Japanese children, and 271/930 of Japanese adolescents) had only one OROS-MPH prescription (Tables 2 and 3). In the US cohort, 21.1% of children and 14.0% of adolescents underwent dose titration once or twice, and 9.5% of children and 4.3% of adolescents underwent titration three or more times. In the Japanese cohort, 21.8% of children and 24.6% of adolescents titrated once or twice, and about 14% of both age groups titrated three or more times. The median duration of the index episodes lengthened with the number of dose titrations.

**Table 3:** Numbers of OROS-MPH dose titration and the corresponding index episode duration, by age groups

	United States		Japan	
	Child group (***n*** = 98,973)	Adolescent group (***n*** = 62,002)	Child group (***n*** = 4595)	Adolescent group (***n*** = 1508)
**Number of dose titration, n (%)**				
0	68,736 (69.4)	50,637 (81.7)	2955 (64.3)	930 (61.7)
≥ 1	30,237 (30.6)	11,365 (18.3)	1640 (35.7)	578 (38.3)
1	13,589 (13.7)	6045 (9.7)	664 (14.5)	242 (16.0)
2	7294 (7.4)	2667 (4.3)	336 (7.3)	130 (8.6)
≥ 3	9354 (9.5)	2653 (4.3)	640 (13.9)	206 (13.7)
**Median duration of the index episode, months (IQR)**				
Patients with 0 dose titration	5.4 (1.0–15.0)	4.8 (1.0–13.4)	7.5 (1.0–21.0)	3.0 (1.0–13.9)
Patients with 1 dose titration	16.4 (8.5–27.3)	13.0 (6.2–21.9)	20.2 (8.8–34.8)	15.3 (5.4–25.5)
Patients with 2 dose titrations	20.6 (12.4–33.2)	16.2 (9.3–26.3)	26.2 (14.0–45.4)	15.7 (7.8–23.4)
Patients with ≥3 dose titrations	28.7 (16.9–46.3)	23.0 (14.0–34.4)	34.2 (18.9–54.5)	23.1 (14.1–37.3)

*OROS-MPH* Osmotic release oral system methylphenidate, *IQR* Inter-quartile range, Child group: 6 to < 13 years, adolescent group: 13 to < 18 years

**Fig. 2:**
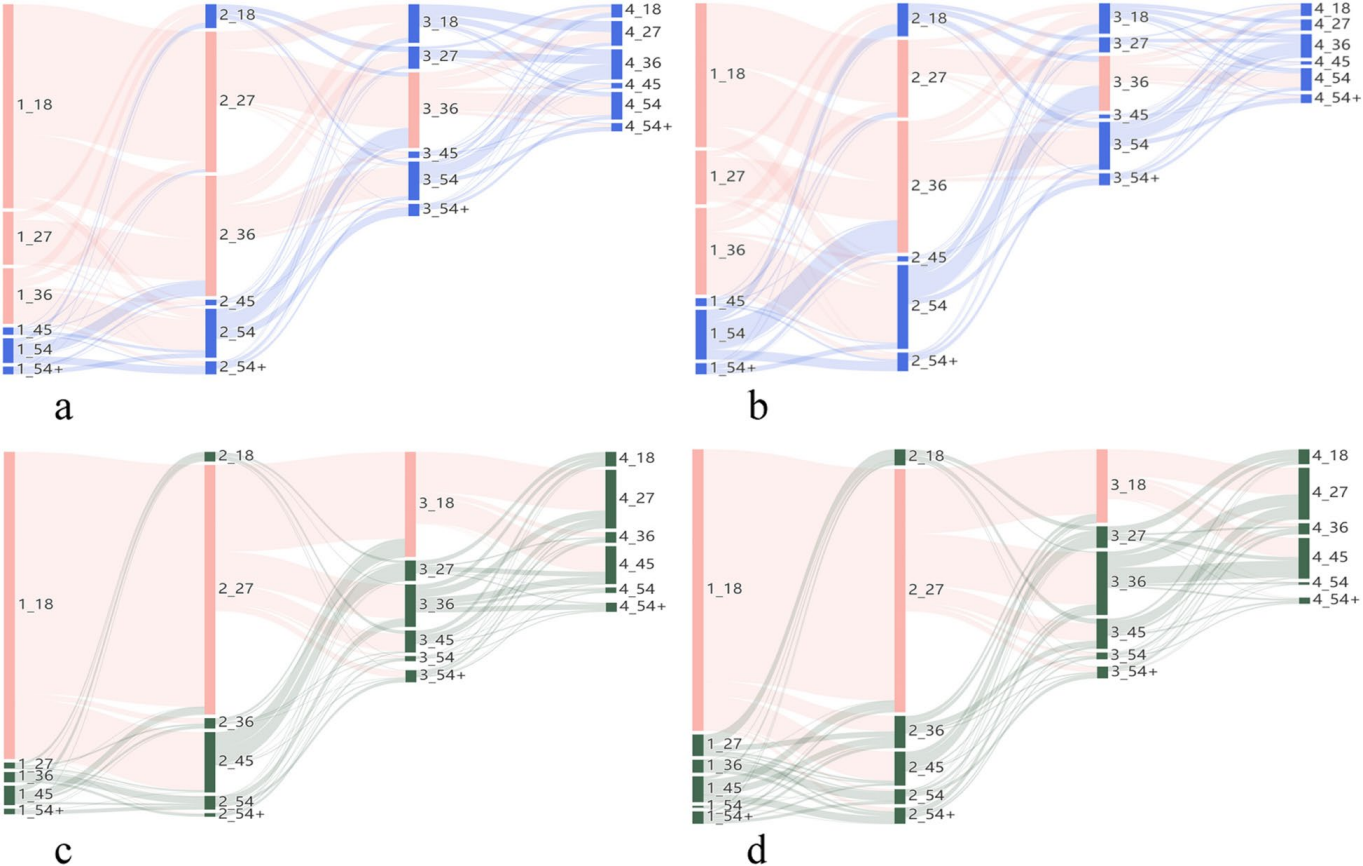
Sankey diagrams of OROS-MPH dose titration patterns among ADHD patients with at least one titration: **a** Child OROS-MPH users in the US cohort (*N* = 30,237); **b** Adolescent OROS-MPH users in the US cohort (*N* = 11,365); **c** Child OROS-MPH users in the Japanese cohort (*N* = 1640); **d** Adolescent OROS-MPH users in the Japanese cohort (*N* = 578). ‘1_18’ refers to patients whose initial OROS-MPH daily dose level was 18 mg/day, ‘2_27’ refers to patients whose second OROS-MPH daily dose level was 27 mg/day, and so on

Titration pathways for patients who underwent at least one dose titration are shown in Sankey diagrams (Fig. 2a-d), derived from data in Supplementary Table 1–4 (Additional file 1).

#### US child group

In the US child group (Fig. 2a & Supplementary Table 1), 30,237 patients underwent dose titration, of whom 84.4% increased initial dose and 15.6% decreased initial dose. Among patients who initiated OROS-MPH at 18, 27, 36 or 45 mg/day, upward titrations (e.g., 18➔27 mg/day, 18➔36 mg/day and 27➔36 mg/day) were more observed than downward titrations (e.g., 27➔18 mg/day and 36➔27 mg/day). However, the opposite was observed among patients who initiated OROS-MPH at ≥54 mg/day. At the second dose level, 55.1% (16,648/30,237) of patients further titrated to a third dose level, of whom 59.3% had a dose increase and 40.7% had a dose decrease. Among these patients, upward titrations still predominated amongst those whose second dose level was 18, 27 or 36 mg/day, while downward titrations predominated in patients whose second dose level was ≥45 mg/day. At the third dose level, 56.2% (9354/16,648) of patients titrated to a fourth dose level, of whom 57.7% had a dose increase and 42.3% had a dose decrease. Among these patients there was a continuing pattern of dose increases in patients taking lower doses and dose decreases in patients at higher doses.

#### US adolescent group

In the US adolescent group (Fig. 2b & Supplementary Table 2), 11,365 patients underwent dose titration, of whom 75.4% increased initial dose and 24.6% decreased initial dose. At the second dose level, 46.8% (5320/11,365) of patients further titrated to a third dose level, of whom 56.2% had a dose increase and 43.8% had a dose decrease. At the third dose level, 49.9% (2653/5320) of patients further titrated to a fourth dose level, of whom 53.0% had a dose increase and 47.0% had a dose decrease. At each dose level, titration patterns were similar to the US child group, with upward titration predominating in patients receiving ≤36 mg/day and downward titration predominating in patients taking higher doses.

#### Japanese child group

In the Japanese child group (Fig. 2c & Supplementary Table 3), 1640 patients underwent dose titration, of whom 92.9% increased their initial dose and 7.1% decreased their initial dose. In this population, 88.2% (1447/1640) initiated OROS-MPH at 18 mg/day, among which 78.7% (1139/1447) increased to 27 mg/day as their second dose level, and, of note, 18.9% (274/1447) directly jumped to 45 mg/day. At the second dose level, 59.5% (976/1640) of this population further titrated to a third dose level, of whom 35.3% had a dose increase and 64.7% had a dose decrease. Downward titrations were commonly 27➔18 mg/day and 45➔18 or 27 mg/day and upward titrations were commonly 27➔36 or 45 mg/day. At the third dose level, 65.6% (640/976) of patients further titrated to a fourth dose level, of whom 71.7% had a dose increase and 28.3% had a dose decrease. Patients whose third dose level was 18 mg/day tended to further increase to 27 or 45 mg/day, and those whose third dose level was 27 or 36 mg/day titrated in either direction.

#### Japanese adolescent group

In the Japanese adolescent group (Fig. 2d & Supplementary Table 4), 578 patients underwent dose titration, of whom 87.7% increased initial dose and 12.3% decreased initial dose. In this population, 79.1% (457/578) initiated OROS-MPH at 18 mg/day, among which 82.3% (376/457) increased to 27 mg/day. At the second dose level, 58.1% (336/578) of patients further titrated to a third dose level, of whom 49.4% had a dose increase and 50.6% had a dose decrease. And at the third dose level, 61.3% (206/336) of patients had further dose titration, of whom 62.1% had a dose increase and 37.9% had a dose decrease. Among the patients at a second or third dose level of 27 or 36 mg/day, a similar proportion of patients further titrated upwards or downwards. Patients at a dose level of ≥45 mg/day were more likely to reduce the daily dose than increase it.

### Sensitivity analyses

In the US cohort, 75,407 children and 49,087 adolescents were MPH-naïve, among which 57.8% of children and 32.9% of adolescents initiated OROS-MPH from the lowest dose (18 mg/day). However, among the patients who were not MPH-native, only 35.7% of children and 19.3% of adolescents initiated OROS-MPH from 18 mg/day. On the other hand, among MPH-naive patients, 31.7% of children and 19.4% of adolescents underwent OROS-MPH dose titration, and their titration pathways were similar to the original populations. Sankey diagrams for sensitivity analyses of MPH-naive children and adolescents in the US cohort were shown in Supplementary Fig. 1a &1b (Additional file 1) respectively, derived from data in Supplementary Table 5 & 6 (Additional file 1).

## Discussion

This retrospective cohort study described OROS-MPH treatment and titration patterns among child and adolescent populations with ADHD in the US and Japan covering study periods of 20 and 12 years respectively. Overall, 160,975 individuals in the US cohort and 6103 individuals in Japanese cohort were included.

Although the US and Japanese cohorts were similar in terms of age and gender, patients’ ADHD medication experiences prior to commencing OROS-MPH were markedly different, reflecting drug availability in each country. In the US, generic MPH had been widely used before OROS-MPH availability, however, no psychostimulants were approved for ADHD indications in Japan until the approval of OROS-MPH in 2007 [27, 33]. Baseline MPH use affects OROS-MPH initial dose. Drug switching from IR to ER MPH (including OROS-MPH) were observed among prevalent MPH users [34]. The approved labels for OROS-MPH [29, 30] in both countries recommend that patients already receiving MPH initiate OROS-MPH based on current dose regimen and clinical judgment. A study of 6–18 years old patients who switched from IR to OROS-MPH [35] reported that higher than equivalent doses of OROS-MPH might be needed for successful drug switching. This is consistent with our observation of a higher starting OROS-MPH dose in children and adolescents who had previous MPH exposure versus MPH-naïve patients.

Over 90% of patients in our study initiated OROS-MPH as monotherapy, especially for the US cohort in which the proportion of monotherapy was as high as 97%, which is in line with recommendations from major ADHD clinical guidelines [16, 18]. In the Japanese cohort, the percentage of patients receiving combination therapy was 8–10%, and nearly 100% (508/510) of these patients received concomitant non-psychostimulants. Combination of psychostimulant and non-psychostimulant might be considered to improve effectiveness of single psychostimulant therapy or when psychostimulant use was limited by side effects [18], however, published evidence was still limited [36].

We observed difference in the treatment patterns employed in the US and Japanese cohorts. In this study, US children and adolescents generally received higher average daily doses of OROS-MPH than the equivalent age groups in Japan. Contributing factors could include previous MPH exposure amongst US patients for which higher OROS-MPH doses are recommended [35, 37]; a higher level of clinical experience using MPH amongst US clinicians versus Japanese clinicians [37]; or higher body weights in US versus Japanese children and adolescents [38, 39]. Body weight is one of the determinants of drug dose [28] and higher body weight has been associated with better tolerability of high MPH doses [40]. Additionally, MPH had been regulated by a third-party committee in Japan since 2008, which could lead to stricter dose control of OROS-MPH in Japan [27, 33].

In our study populations, the maximum daily dose in the US cohort was higher than the Japanese cohort. Recommended maximum dose of OROS-MPH was derived from clinical trials, but has not been fully justified [41]. The US drug label [29] recommends an upper limit of 54 mg/day OROS-MPH for children and 72 mg/day for adolescents, while the Japanese label [30] recommends 54 mg/day as the upper limit for both children and adolescents. In our study, we observed that the approved maximum dose was exceeded by 4.6% of children and by 11.6% of adolescents in the Japanese cohort. And in another study using the same Japanese database [27], the maximum dose was exceeded by 11.6% of patients aged 6–65 years. These findings indicated that overdose of OROS-MPH was not rare and there might be a positive association between age and overdose. Some authors have questioned the appropriateness of setting upper does limits, arguing that a small group of patients may benefit from higher-than-approved doses without serious adverse consequences [41].

According to the prescription information, the majority (> 60%) of patients in this study received OROS-MPH only at an initial dose level. In the Japanese cohort, the proportions of patients didn’t undergo titration were similar in children and adolescents, whereas, in the US cohort, more adolescents didn’t undergo dose titration than children. Among these patients without dose titration, approximately 30% had only one OROS-MPH prescription. ADHD patients who failed to file a second medication prescription could be defined as early cessation of therapy indicating intolerable side-effects [42, 43]. On the other hand, the other 70% of patients without dose titration had two or more prescriptions at the initial dose level, which might indicate acceptable treatment response at this dose level without requiring dose adjustments.

OROS-MPH titration patterns were different between the US and Japanese cohorts in real-world settings. Compared with patients in the US cohort, Japanese patients had more numbers of dose titration, with 14% of Japanese children and adolescents having at least three titrations, versus 9.5% of children and 4.3% of adolescents in the US cohort. Most Japanese patients initiated OROS-MPH at the lowest dose (18 mg/day), whereas US children had a broader distribution of initial doses (18, 27, 36 and 54 mg/day). From the initial to the second dose level, patients in both countries tended to titrate upwards. Form the second to the third dose level, however, different titration trends were observed. US patients at a second dose level of 27 or 36 mg/day tended to continue increasing their dose, whereas a half of Japanese patients at a second dose level of 27 mg/day decreased their dose back to 18 mg/day. US patients most often maintained OROS-MPH at 36 mg/day, versus 18 mg/day for Japanese patients. Results from the sensitivity analyses indicated that prior generic MPH experience didn’t influence OROS-MPH titration patterns, although it could affect the initial OROS-MPH dose.

In this study, dose titrations were usually made in increments of 9 or 18 mg, which was consistent with the drug labels [29, 30]. However, among Japanese children who initiated OROS-MPH from 18 mg/day and underwent titration, 18.9% directly jumped to 45 mg/day as their second dose level, which went against the label. Although we had no definite explanation for this result, we found that long-term OROS-MPH users (i.e., patients who initiated OROS-MPH in childhood and continuously used it into their adolescence) among this population accounted for a larger proportion than among other populations, and the high daily dose often occurred during their adolescence. This suggested that long-term use of OROS-MPH might be associated with higher dose level.

So far as we know, this is the first real-world study describing detailed titration patterns of OROS-MPH. Strengths of the study include the use of two large claims databases in countries with different experience using MPH, diverse ethnic and cultural characteristics, different healthcare structures that make our findings generalisable to Western and Eastern countries; and an extended study period covering 20 years for the US cohort and 12 years for the Japanese cohort, allowing capture of OROS-MPH patterns of use since drug approval in each country. The findings of this study can be used to support clinical decision making and particularly of great reference for developing countries with a large number of ADHD patients but insufficient experience in OROS-MPH use.

Potential study limitations are that the claims databases only include reported and billed insurance records, and the data may be subject to potential coding mistakes and inaccuracies, hence the findings may not be completely representative of the entire population of patients with ADHD in these countries. The claims databases captured prescription information, not whether the medication was taken as prescribed and the actual titration patterns may not be fully reflected by drug prescriptions [31]. In the US and Japan, OROS-MPH was approved at different times and in different ADHD treatment conditions, and these differences should be considered in the comparisons of the results between two countries. Finally, the claims databases are designed for reimbursement purposes and do no capture effectiveness endpoints (such as ADHD rating scores) and safety endpoints (such as adverse events), therefore the underlying reasons of dose adjustment could not be confirmed.

## Conclusions

In conclusion, our study described real-world OROS-MPH treatment patterns in patients with ADHD in the US and Japan. While we observed some differences in drug use and dose titration patterns in the two countries, the majority of patients were treated according to recommendations provided in the product labels, suggesting that these recommendations are relevant in real world clinical practice. Additional real-world studies could provide information about clinical reasoning underlying dose selection and titration patterns, and would provide useful information to support clinical decisions.

## Supplementary Information

**Additional file 1: Supplementary Table 1.** Numbers of OROS-MPH users at each dose level among patients with at least one dose titration (Data for Fig. 2a: child group in the US cohort, *N* = 30,237). **Supplementary Table 2.** Numbers of OROS-MPH users at each dose level among patients with at least one dose titration (Data for Fig. 2b: adolescent group in the US cohort, *N* = 11,365). **Supplementary Table 3.** Numbers of OROS-MPH users at each dose level among patients with at least one dose titration (Data for Fig. 2c: child group in the Japanese cohort, *N* = 1640). **Supplementary Table 4.** Numbers of OROS-MPH users at each dose level among patients with at least one dose titration (Data for Fig. 2d: adolescent group in the Japanese cohort, *N* = 578). **Supplementary Table 5.** Numbers of OROS-MPH users at each dose level among MPH-naïve patients with at least one dose titration (Data for Supplementary Fig. 1a: child group of sensitivity analysis in the US cohort, *N* = 23,901). **Supplementary Table 6.** Numbers of OROS-MPH users at each dose level among MPH-naïve patients with at least one dose titration (Data for Supplementary Fig. 1b: adolescent group of sensitivity analysis in the US cohort, *N* = 9512). **Supplementary Fig. 1.** Sensitivity analysis: Sankey diagrams of OROS-MPH dose titration patterns among MPH-naïve ADHD patients with at least one titration: Supplementary Fig. 1a Child OROS-MPH users in the US cohort (N = 23,901); Supplementary Fig. 1b Adolescent OROS-MPH users in the US cohort (N = 9512). ‘1_18’ refers to patients whose initial OROS-MPH daily dose level was 18 mg/day, ‘2_27’ refers to patients whose second OROS-MPH daily dose level was 27 mg/day, and so on.

## Data Availability

The data that support the findings of this study are available from IMB Watson Health and JMDC Inc. but restrictions apply to the availability of these data, which were used under license for the current study, and so are not publicly available. Data are however available from the authors upon reasonable request and with permission of IMB Watson Health and JMDC Inc..

## Abbreviations

ADHD: Attention-deficit hyperactivity disorder
CCAE: IBM® MarketScan® Commercial Claims and Encounters
ER: Extended release
ICD-10-CM: International Classification of Diseases, Tenth Revision, Clinical Modification
ICD-9-CM: International Classification of Diseases, Ninth Revision, Clinical Modification
IR: Immediate release
JMDC: Japan Medical Data Centre
MPH: Methylphenidate
NICE: National Institute for Health and Care Excellence
OROS-MPH: Osmotic release oral system methylphenidate

## References

[1] Cortese S, Adamo N, Del Giovane C, Mohr-Jensen C, Hayes AJ, Carucci S (2018). Comparative efficacy and tolerability of medications for attention-deficit hyperactivity disorder in children, adolescents, and adults: a systematic review and network meta-analysis. Lancet Psychiatry.

[2] Posner J, Polanczyk GV, Sonuga-Barke E (2020). Attention-deficit hyperactivity disorder. Lancet..

[3] Polanczyk GV, Salum GA, Sugaya LS, Caye A, Rohde LA (2015). Annual research review: a meta-analysis of the worldwide prevalence of mental disorders in children and adolescents. J Child Psychol Psychiatry Allied Discip.

[4] Polanczyk G, De Lima MS, Horta BL, Biederman J, Rohde LA (2007). The worldwide prevalence of ADHD: a systematic review and metaregression analysis. Am J Psychiatry.

[5] Thomas R, Sanders S, Doust J, Beller E, Glasziou P (2015). Prevalence of attention- deficit/hyperactivity disorder: a systematic review and meta-analysis. Pediatrics..

[6] Willcutt EG (2012). The prevalence of DSM-IV attention-deficit/hyperactivity disorder: a meta-analytic review. Neurotherapeutics..

[7] Asherson P, Agnew-Blais J (2019). Annual research review: does late-onset attention-deficit/hyperactivity disorder exist?. J Child Psychol Psychiatry.

[8] Krasner AJ, Turner JB, Feldman JF, Silberman AE, Fisher PW, Workman CC (2018). ADHD symptoms in a non-referred low birthweight/preterm cohort-supplement. J Atten Disord.

[9] Sibley MH, Rohde LA, Swanson JM, Hechtman LT, Molina BSG, Mitchell JT (2018). Late-onset ADHD reconsidered with comprehensive repeated assessments between ages 10 and 25. Am J Psychiatry.

[10] Moffitt TE, Houts R, Asherson P, Belsky DW, Corcoran DL, Hammerle M (2015). Is adult ADHD a childhood-onset neurodevelopmental disorder? Evidence from a four-decade longitudinal cohort study. Am J Psychiatry.

[11] Polanczyk GV, Willcutt EG, Salum GA, Kieling C, Rohde LA (2014). ADHD prevalence estimates across three decades: an updated systematic review and meta-regression analysis. Int J Epidemiol.

[12] Wang T, Liu K, Li Z, Xu Y, Liu Y, Shi W (2017). Prevalence of attention deficit/hyperactivity disorder among children and adolescents in China: a systematic review and meta-analysis. BMC Psychiatry.

[13] Liu A, Xu Y, Yan Q, Tong L (2018). The prevalence of attention deficit/hyperactivity disorder among Chinese children and adolescents. Sci Rep.

[14] Raman SR, Man KKC, Bahmanyar S, Berard A, Bilder S, Boukhris T (2018). Trends in attention-deficit hyperactivity disorder medication use: a retrospective observational study using population-based databases. Lancet Psychiatry.

[15] Bolea-Alamañac B, Nutt DJ, Adamou M, Asherson P, Bazire S, Coghill D (2014). Evidence-based guidelines for the pharmacological management of attention deficit hyperactivity disorder: update on recommendations from the British Association for Psychopharmacology. J Psychopharmacol.

[16] National Institute for Health and Care Excellence (NICE) (2018). Attention deficit hyperactivity disorder: diagnosis and management. NICE Clinical Guideline CG87.

[17] Wolraich M, Brown L, Brown RT, DuPaul G, Subcommittee on Attention-Deficit/Hyperactivity Disorder, Steering Committee on Quality Improvement and Management (2011). ADHD: clinical practice guideline for the diagnosis, evaluation and treatment of attention-deficit/hyperactivity disorder in children and adolescents. Pediatrics..

[18] Wolraich ML, Hagan JF, Allan C, Chan E, Davison D, Earls M (2019). Clinical practice guideline for the diagnosis, evaluation, and treatment of attention-deficit/hyperactivity disorder in children and adolescents. Pediatrics..

[19] Hodgkins P, Setyawan J, Mitra D, Davis K, Quintero J, Fridman M (2013). Management of ADHD in children across Europe: patient demographics, physician characteristics and treatment patterns. Eur J Pediatr.

[20] Furu K, Karlstad Ø, Zoega H, Martikainen JE, Bahmanyar S, Kieler H (2017). Utilization of stimulants and atomoxetine for attention-deficit/hyperactivity disorder among 5.4 million children using population-based longitudinal data. Basic Clin Pharmacol Toxicol.

[21] Katzman MA, Sternat T (2014). A review of OROS methylphenidate (Concerta®) in the treatment of attention-deficit/hyperactivity disorder. CNS Drugs.

[22] Gau SSF, Shen HY, Soong WT, Gau CS (2006). An open-label, randomized, active-controlled equivalent trial of osmotic release oral system methylphenidate in children with attention-deficit/ hyperactivity disorder in Taiwan. J Child Adolesc Psychopharmacol.

[23] Pelham WE, Gnagy EM, Burrows-Maclean L, Williams A, Fabiano GA, Morrisey SM (2001). Once-a-day Concerta methylphenidate versus three-times-daily methylphenidate in laboratory and natural settings. Pediatrics..

[24] Ponizovsky AM, Marom E, Fitoussi I (2014). Trends in attention deficit hyperactivity disorder drugs consumption, Israel, 2005-2012. Pharmacoepidemiol Drug Saf.

[25] Štuhec M, Locatelli I, Švab V (2015). Trends in attention-deficit/hyperactivity disorder drug consumption in children and adolescents in Slovenia from 2001 to 2012: a drug use study from a national perspective. J Child Adolesc Psychopharmacol.

[26] Okumura Y, Usami M, Okada T, Saito T, Negoro H, Tsujii N (2018). Prevalence, incidence and persistence of ADHD drug use in Japan. Epidemiol Psychiatr Sci.

[27] Fife D, Voss EA, Hardin J, Rofael H, Solomon ID, Ryan PB (2021). Medications for attention-deficit/hyperactivity disorder in Japan: a retrospective cohort study of label compliance. Neuropsychopharmacol Rep.

[28] Chermá MD, Josefsson M, Rydberg I, Woxler P, Trygg T, Hollertz O (2017). Methylphenidate for treating ADHD: a naturalistic clinical study of methylphenidate blood concentrations in children and adults with optimized dosage. Eur J Drug Metab Pharmacokinet.

[29] US Food and Drug Administration (FDA) (2021). CONCERTA-methylphenidate hydrochloride tablet, extended release.

[30] Japan Pharmaceuticals and Medical Devices Agency (PMDA) (2021). CONCERTA® Tablets Prolonged-release tablets of methylphenidate hydrochloride.

[31] Brinkman WB, Epstein JN (2011). Promoting productive interactions between parents and physicians in the treatment of children with attention-deficit/hyperactivity disorder. Expert Rev Neurother.

[32] Epstein JN, Rabiner D, Johnson DE, Fitzgerald DP, Chrisman A, Erkanli A (2007). Improving attention-deficit/hyperactivity disorder treatment outcomes through use of a collaborative consultation treatment service by community-based pediatricians: a cluster randomized trial. Arch Pediatr Adolesc Med.

[33] Yoshida M, Obara T, Kikuchi S, Satoh M, Morikawa Y, Ooba N (2020). Drug prescriptions for children with ADHD in Japan: a study based on health insurance claims data between 2005 and 2015. J Atten Disord.

[34] Karlstad Ø, Zoëga H, Furu K, Bahmanyar S, Martikainen JE, Kieler H (2016). Use of drugs for ADHD among adults-a multinational study among 15.8 million adults in the Nordic countries. Eur J Clin Pharmacol.

[35] Gormez V, Avery B, Mann H (2013). Switching from immediate release to sustained release methylphenidate in the treatment of children and adolescents with attention deficit/hyperactivity disorder. Eur Rev Med Pharmacol Sci.

[36] Treuer T, Gau SSF, Méndez L, Montgomery W, Monk JA, Altin M (2013). A systematic review of combination therapy with stimulants and atomoxetine for attention-deficit/hyperactivity disorder, including patient characteristics, treatment strategies, effectiveness, and tolerability. J Child Adolesc Psychopharmacol.

[37] Kemner JE, Starr HL, Ciccone PE, Hooper-Wood CG, Crockett RS (2005). Outcomes of OROS® methylphenidate compared with atomoxetine in children with ADHD: a multicenter, randomized prospective study. Adv Ther.

[38] Skinner AC, Ravanbakht SN, Skelton JA, Perrin EM, Armstrong SC (2018). Prevalence of obesity and severe obesity in US children, 1999-2016. Pediatrics..

[39] Ochiai H, Shirasawa T, Nishimura R, Yoshimoto T, Minoura A, Oikawa K (2020). Changes in overweight/obesity and central obesity status from preadolescence to adolescence: a longitudinal study among schoolchildren in Japan. BMC Public Health.

[40] Becker SP, Froehlich TE, Epstein JN (2016). Effects of methylphenidate on sleep functioning in children with attention-deficit/hyperactivity disorder. J Dev Behav Pediatr.

[41] Ching C, Eslick GD, Poulton AS (2019). Evaluation of methylphenidate safety and maximum-dose titration rationale in attention-deficit/hyperactivity disorder: a meta-analysis. JAMA Pediatr.

[42] Pottegård A, Bjerregaard BK, Glintborg D, Kortegaard LS, Hallas J, Moreno SI (2013). The use of medication against attention deficit/hyperactivity disorder in Denmark: a drug use study from a patient perspective. Eur J Clin Pharmacol.

[43] Pottegård A, Bjerregaard BK, Kortegaard LS, Zoëga H (2015). Early discontinuation of attention-deficit/hyperactivity disorder drug treatment: a Danish nationwide drug utilization study. Basic Clin Pharmacol Toxicol.

[44] United States Congress (2010). Patient Protection and Affordable Care Act.

[45] Ministry of Education, Culture, Sports, Science and Technology, Ministry of Health, Labour and Welfare, and Ministry of Economy, Trade and Industry (2014). Ethical guidelines for medical and biological research involving human subjects.

