# Supplementary material for: Dose titration of osmotic release oral system methylphenidate in children and adolescents with attention-deficit hyperactivity disorder: a retrospective cohort study
Source: BMC Pediatr. 2023 Jan 23;23:38. doi: 10.1186/s12887-023-03850-4 (PMC9869580; doi:10.1186/s12887-023-03850-4)
Supplement: Supplementary file 1 — Additional file 1: Supplementary Table 1. Numbers of OROS-MPH users at each dose level among patients with at least one dose titration (Data for Fig. 2a: child group in the US cohort, N = 30,237). Supplementary Table 2. Numbers of OROS-MPH users at each dose level among patients with at least one dose titration (Data for Fig. 2b: adolescent group in the US cohort, N = 11,365). Supplementary Table 3. Numbers of OROS-MPH users at each dose level among patients with at least one dose titration (Data for Fig. 2c: child group in the Japanese cohort, N = 1640). Supplementary Table 4. Numbers of OROS-MPH users at each dose level among patients with at least one dose titration (Data for Fig. 2d: adolescent group in the Japanese cohort, N = 578). Supplementary Table 5. Numbers of OROS-MPH users at each dose level among MPH-naïve patients with at least one dose titration (Data for Supplementary Fig. 1a: child group of sensitivity analysis in the US cohort, N = 23,901). Supplementary Table 6. Numbers of OROS-MPH users at each dose level among MPH-naïve patients with at least one dose titration (Data for Supplementary Fig. 1b: adolescent group of sensitivity analysis in the US cohort, N = 9512). Supplementary Fig. 1. Sensitivity analysis: Sankey diagrams of OROS-MPH dose titration patterns among MPH-naïve ADHD patients with at least one titration: Supplementary Fig. 1a Child OROS-MPH users in the US cohort (N = 23,901); Supplementary Fig. 1b Adolescent OROS-MPH users in the US cohort (N = 9512). ‘1_18’ refers to patients whose initial OROS-MPH daily dose level was 18 mg/day, ‘2_27’ refers to patients whose second OROS-MPH daily dose level was 27 mg/day, and so on. [file 12887_2023_3850_MOESM1_ESM.docx]

**Supplementary Information**

**Supplementary Table 1.** Numbers of OROS-MPH users at each dose level among patients with at least one dose titration (Data for Fig. 2a: child group in the US cohort, N=30,237)

| **1st to 2nd daily dose level** | | | | | | | | |
| --- | --- | --- | --- | --- | --- | --- | --- | --- |
| **Dose 1, md/day** | **Dose 2, md/day** | **Titrated patients** | **Upward titration, %** | | **Downward titration, %** | | **No further titration, %** | |
| **18 (N=17,524)** | -- | -- | 17,524 | 100.0% | 0 | 0.0% | -- | -- |
|  | 27 | 11,153 |  |  |  |  |  |  |
|  | 36 | 5,398 |  |  |  |  |  |  |
|  | 45 | 126 |  |  |  |  |  |  |
|  | 54 | 795 |  |  |  |  |  |  |
|  | >54 | 52 |  |  |  |  |  |  |
| **27 (N=4,542)** | 18 | 689 | 3,853 | 84.8% | 689 | 15.2% | -- | -- |
|  | -- | -- |  |  |  |  |  |  |
|  | 36 | 3,594 |  |  |  |  |  |  |
|  | 45 | 50 |  |  |  |  |  |  |
|  | 54 | 160 |  |  |  |  |  |  |
|  | >54 | 49 |  |  |  |  |  |  |
| **36 (N=4,775)** | 18 | 909 | 3,175 | 66.5% | 1,600 | 33.5% | -- | -- |
|  | 27 | 691 |  |  |  |  |  |  |
|  | -- | -- |  |  |  |  |  |  |
|  | 45 | 284 |  |  |  |  |  |  |
|  | 54 | 2,597 |  |  |  |  |  |  |
|  | >54 | 294 |  |  |  |  |  |  |
| **45 (N=623)** | 18 | 71 | 347 | 55.7% | 276 | 44.3% | -- | -- |
|  | 27 | 115 |  |  |  |  |  |  |
|  | 36 | 90 |  |  |  |  |  |  |
|  | -- | -- |  |  |  |  |  |  |
|  | 54 | 245 |  |  |  |  |  |  |
|  | >54 | 102 |  |  |  |  |  |  |
| **54 (N=2,118)** | 18 | 355 | 614 | 29.0% | 1,504 | 71.0% | -- | -- |
|  | 27 | 87 |  |  |  |  |  |  |
|  | 36 | 1,042 |  |  |  |  |  |  |
|  | 45 | 20 |  |  |  |  |  |  |
|  | -- | -- |  |  |  |  |  |  |
|  | >54 | 614 |  |  |  |  |  |  |
| **>54 (N=655)** | 18 | 21 | 0 | 0.0% | 655 | 100.0% |  |  |
|  | 27 | 24 |  |  |  |  |  |  |
|  | 36 | 210 |  |  |  |  |  |  |
|  | 45 | 3 |  |  |  |  |  |  |
|  | 54 | 397 |  |  |  |  |  |  |
|  | -- | -- |  |  |  |  |  |  |
| **2nd to 3rd daily dose level** | | | | | | | | |
| **Dose 2, md/day** | **Dose 3, mg/day** | **Titrated patients** | **Upward titration, %** | | **Downward titration, %** | | **No further titration, %** | |
| **18 (N=2,045)** | -- | -- | 1,249 | 61.1% | 0 | 0.0% | 796 | 38.9% |
|  | 27 | 490 |  |  |  |  |  |  |
|  | 36 | 369 |  |  |  |  |  |  |
|  | 45 | 25 |  |  |  |  |  |  |
|  | 54 | 320 |  |  |  |  |  |  |
|  | >54 | 45 |  |  |  |  |  |  |
| **27 (N=12,070)** | 18 | 1,585 | 4,739 | 39.3% | 1,585 | 13.1% | 5,746 | 47.6% |
|  | -- | -- |  |  |  |  |  |  |
|  | 36 | 4,401 |  |  |  |  |  |  |
|  | 45 | 139 |  |  |  |  |  |  |
|  | 54 | 150 |  |  |  |  |  |  |
|  | >54 | 49 |  |  |  |  |  |  |
| **36 (N=10,334)** | 18 | 1,305 | 3,056 | 29.6% | 2,482 | 24.0% | 4,796 | 46.4% |
|  | 27 | 1,177 |  |  |  |  |  |  |
|  | -- | -- |  |  |  |  |  |  |
|  | 45 | 332 |  |  |  |  |  |  |
|  | 54 | 2,499 |  |  |  |  |  |  |
|  | >54 | 225 |  |  |  |  |  |  |
| **45 (N=483)** | 18 | 54 | 96 | 19.9% | 194 | 40.2% | 193 | 40.0% |
|  | 27 | 97 |  |  |  |  |  |  |
|  | 36 | 43 |  |  |  |  |  |  |
|  | -- | -- |  |  |  |  |  |  |
|  | 54 | 91 |  |  |  |  |  |  |
|  | >54 | 5 |  |  |  |  |  |  |
| **54 (N=4,194)** | 18 | 345 | 731 | 17.4% | 1,855 | 44.2% | 1,608 | 38.3% |
|  | 27 | 119 |  |  |  |  |  |  |
|  | 36 | 1,347 |  |  |  |  |  |  |
|  | 45 | 44 |  |  |  |  |  |  |
|  | -- | -- |  |  |  |  |  |  |
|  | >54 | 731 |  |  |  |  |  |  |
| **>54 (N=1,111)** | 18 | 25 | 0 | 0.0% | 661 | 59.5% | 450 | 40.5% |
|  | 27 | 34 |  |  |  |  |  |  |
|  | 36 | 334 |  |  |  |  |  |  |
|  | 45 | 4 |  |  |  |  |  |  |
|  | 54 | 264 |  |  |  |  |  |  |
|  | -- | -- |  |  |  |  |  |  |
| **3rd to 4th daily dose level** | | | | | | | | |
| **Dose 3, md/day** | **Dose 4, mg/day** | **Titrated patients** | **Upward titration, %** | | **Downward titration, %** | | **No further titration, %** | |
| **18 (N=3,314)** | -- | -- | 1,955 | 59.0% | 0 | 0.0% | 1,359 | 41.0% |
|  | 27 | 942 |  |  |  |  |  |  |
|  | 36 | 568 |  |  |  |  |  |  |
|  | 45 | 73 |  |  |  |  |  |  |
|  | 54 | 327 |  |  |  |  |  |  |
|  | >54 | 45 |  |  |  |  |  |  |
| **27 (N=1,917)** | 18 | 232 | 846 | 44.1% | 232 | 12.1% | 839 | 43.8% |
|  | -- | -- |  |  |  |  |  |  |
|  | 36 | 696 |  |  |  |  |  |  |
|  | 45 | 55 |  |  |  |  |  |  |
|  | 54 | 64 |  |  |  |  |  |  |
|  | >54 | 31 |  |  |  |  |  |  |
| **36 (N=6,494)** | 18 | 499 | 2,135 | 32.9% | 1,413 | 21.8% | 2,946 | 45.4% |
|  | 27 | 914 |  |  |  |  |  |  |
|  | -- | -- |  |  |  |  |  |  |
|  | 45 | 277 |  |  |  |  |  |  |
|  | 54 | 1,593 |  |  |  |  |  |  |
|  | >54 | 265 |  |  |  |  |  |  |
| **45 (N=544)** | 18 | 53 | 113 | 20.8% | 231 | 42.5% | 200 | 36.8% |
|  | 27 | 120 |  |  |  |  |  |  |
|  | 36 | 58 |  |  |  |  |  |  |
|  | -- | -- |  |  |  |  |  |  |
|  | 54 | 107 |  |  |  |  |  |  |
|  | >54 | 6 |  |  |  |  |  |  |
| **54 (N=3,324)** | 18 | 329 | 349 | 10.5% | 1,546 | 46.5% | 1,429 | 43.0% |
|  | 27 | 117 |  |  |  |  |  |  |
|  | 36 | 1,049 |  |  |  |  |  |  |
|  | 45 | 51 |  |  |  |  |  |  |
|  | -- | -- |  |  |  |  |  |  |
|  | >54 | 349 |  |  |  |  |  |  |
| **>54 (N=1,055)** | 18 | 21 | 0 | 0.0% | 534 | 50.6% | 521 | 49.4% |
|  | 27 | 28 |  |  |  |  |  |  |
|  | 36 | 205 |  |  |  |  |  |  |
|  | 45 | 3 |  |  |  |  |  |  |
|  | 54 | 277 |  |  |  |  |  |  |
|  | -- | -- |  |  |  |  |  |  |

Child group: 6 to <13 years;

**Supplementary Table 2.** Numbers of OROS-MPH users at each dose level among patients with at least one dose titration (Data for Fig. 2b: adolescent group in the US cohort, N=11,365)

| **1st to 2nd daily dose level** | | | | | | | | |
| --- | --- | --- | --- | --- | --- | --- | --- | --- |
| **Dose 1, md/day** | **Dose 2, md/day** | **Titrated patients** | **Upward titration, %** | | **Downward titration, %** | | **No further titration, %** | |
| **18 (N=4,674)** | -- | -- | 4,674 | 100.0% | 0 | 0.0% | -- | -- |
|  | 27 | 2,042 |  |  |  |  |  |  |
|  | 36 | 1,955 |  |  |  |  |  |  |
|  | 45 | 31 |  |  |  |  |  |  |
|  | 54 | 613 |  |  |  |  |  |  |
|  | >54 | 33 |  |  |  |  |  |  |
| **27 (N=1,641)** | 18 | 203 | 1,438 | 87.6% | 203 | 12.4% | -- | -- |
|  | -- | -- |  |  |  |  |  |  |
|  | 36 | 1,265 |  |  |  |  |  |  |
|  | 45 | 18 |  |  |  |  |  |  |
|  | 54 | 135 |  |  |  |  |  |  |
|  | >54 | 20 |  |  |  |  |  |  |
| **36 (N=2,817)** | 18 | 480 | 2,036 | 72.3% | 781 | 27.7% | -- | -- |
|  | 27 | 301 |  |  |  |  |  |  |
|  | -- | -- |  |  |  |  |  |  |
|  | 45 | 90 |  |  |  |  |  |  |
|  | 54 | 1,776 |  |  |  |  |  |  |
|  | >54 | 170 |  |  |  |  |  |  |
| **45 (N=256)** | 18 | 35 | 111 | 43.4% | 145 | 56.6% | -- | -- |
|  | 27 | 75 |  |  |  |  |  |  |
|  | 36 | 35 |  |  |  |  |  |  |
|  | -- | -- |  |  |  |  |  |  |
|  | 54 | 41 |  |  |  |  |  |  |
|  | >54 | 70 |  |  |  |  |  |  |
| **54 (N=1,614)** | 18 | 332 | 313 | 19.4% | 1,301 | 80.6% | -- | -- |
|  | 27 | 77 |  |  |  |  |  |  |
|  | 36 | 869 |  |  |  |  |  |  |
|  | 45 | 23 |  |  |  |  |  |  |
|  | -- | -- |  |  |  |  |  |  |
|  | >54 | 313 |  |  |  |  |  |  |
| **>54 (N=363)** | 18 | 26 | 0 | 0.0% | 363 | 100.0% | -- | -- |
|  | 27 | 20 |  |  |  |  |  |  |
|  | 36 | 152 |  |  |  |  |  |  |
|  | 45 | 6 |  |  |  |  |  |  |
|  | 54 | 159 |  |  |  |  |  |  |
|  | -- | -- |  |  |  |  |  |  |
| **2nd to 3rd daily dose level** | | | | | | | | |
| **Dose 2, md/day** | **Dose 3, mg/day** | **Titrated patients** | **Upward titration, %** | | **Downward titration, %** | | **No further titration, %** | |
| **18 (N=1,076)** | -- | -- | 570 | 53.0% | 0 | 0.0% | 506 | 47.0% |
|  | 27 | 132 |  |  |  |  |  |  |
|  | 36 | 191 |  |  |  |  |  |  |
|  | 45 | 11 |  |  |  |  |  |  |
|  | 54 | 195 |  |  |  |  |  |  |
|  | >54 | 41 |  |  |  |  |  |  |
| **27 (N=2,515)** | 18 | 256 | 896 | 35.6% | 256 | 10.2% | 1,363 | 54.2% |
|  | -- | -- |  |  |  |  |  |  |
|  | 36 | 775 |  |  |  |  |  |  |
|  | 45 | 25 |  |  |  |  |  |  |
|  | 54 | 76 |  |  |  |  |  |  |
|  | >54 | 20 |  |  |  |  |  |  |
| **36 (N=4,276)** | 18 | 422 | 1,294 | 30.3% | 681 | 15.9% | 2,301 | 53.8% |
|  | 27 | 259 |  |  |  |  |  |  |
|  | -- | -- |  |  |  |  |  |  |
|  | 45 | 66 |  |  |  |  |  |  |
|  | 54 | 1,104 |  |  |  |  |  |  |
|  | >54 | 124 |  |  |  |  |  |  |
| **45 (N=168)** | 18 | 23 | 32 | 19.0% | 50 | 29.8% | 86 | 51.2% |
|  | 27 | 15 |  |  |  |  |  |  |
|  | 36 | 12 |  |  |  |  |  |  |
|  | -- | -- |  |  |  |  |  |  |
|  | 54 | 29 |  |  |  |  |  |  |
|  | >54 | 3 |  |  |  |  |  |  |
| **54 (N=2,724)** | 18 | 264 | 199 | 7.3% | 1,071 | 39.3% | 1,454 | 53.4% |
|  | 27 | 69 |  |  |  |  |  |  |
|  | 36 | 719 |  |  |  |  |  |  |
|  | 45 | 19 |  |  |  |  |  |  |
|  | -- | -- |  |  |  |  |  |  |
|  | >54 | 199 |  |  |  |  |  |  |
| **>54 (N=606)** | 18 | 23 | 0 | 0.0% | 271 | 44.7% | 335 | 55.3% |
|  | 27 | 16 |  |  |  |  |  |  |
|  | 36 | 85 |  |  |  |  |  |  |
|  | 45 | 1 |  |  |  |  |  |  |
|  | 54 | 146 |  |  |  |  |  |  |
|  | -- | -- |  |  |  |  |  |  |
| **3rd to 4th daily dose level** | | | | | | | | |
| **Dose 3, md/day** | **Dose 4, mg/day** | **Titrated patients** | **Upward titration, %** | | **Downward titration, %** | | **No further titration, %** | |
| **18 (N=988)** | -- | -- | 496 | 50.2% | 0 | 0.0% | 492 | 49.8% |
|  | 27 | 132 |  |  |  |  |  |  |
|  | 36 | 158 |  |  |  |  |  |  |
|  | 45 | 27 |  |  |  |  |  |  |
|  | 54 | 144 |  |  |  |  |  |  |
|  | >54 | 35 |  |  |  |  |  |  |
| **27 (N=491)** | 18 | 46 | 177 | 36.0% | 46 | 9.4% | 268 | 54.6% |
|  | -- | -- |  |  |  |  |  |  |
|  | 36 | 114 |  |  |  |  |  |  |
|  | 45 | 14 |  |  |  |  |  |  |
|  | 54 | 30 |  |  |  |  |  |  |
|  | >54 | 19 |  |  |  |  |  |  |
| **36 (N=1,782)** | 18 | 153 | 569 | 31.9% | 286 | 16.0% | 927 | 52.0% |
|  | 27 | 133 |  |  |  |  |  |  |
|  | -- | -- |  |  |  |  |  |  |
|  | 45 | 46 |  |  |  |  |  |  |
|  | 54 | 446 |  |  |  |  |  |  |
|  | >54 | 77 |  |  |  |  |  |  |
| **45 (N=122)** | 18 | 13 | 12 | 9.8% | 52 | 42.6% | 58 | 47.5% |
|  | 27 | 24 |  |  |  |  |  |  |
|  | 36 | 15 |  |  |  |  |  |  |
|  | -- | -- |  |  |  |  |  |  |
|  | 54 | 11 |  |  |  |  |  |  |
|  | >54 | 1 |  |  |  |  |  |  |
| **54 (N=1,550)** | 18 | 184 | 152 | 9.8% | 649 | 41.9% | 749 | 48.3% |
|  | 27 | 58 |  |  |  |  |  |  |
|  | 36 | 394 |  |  |  |  |  |  |
|  | 45 | 13 |  |  |  |  |  |  |
|  | -- | -- |  |  |  |  |  |  |
|  | >54 | 152 |  |  |  |  |  |  |
| **>54 (N=387)** | 18 | 14 | 0 | 0.0% | 214 | 55.3% | 173 | 44.7% |
|  | 27 | 11 |  |  |  |  |  |  |
|  | 36 | 86 |  |  |  |  |  |  |
|  | 45 | 3 |  |  |  |  |  |  |
|  | 54 | 100 |  |  |  |  |  |  |
|  | -- | -- |  |  |  |  |  |  |

Adolescent group: 13 to <18 years

**Supplementary Table 3.** Numbers of OROS-MPH users at each dose level among patients with at least one dose titration (Data for Fig. 2c: child group in the Japanese cohort, N=1,640)

| **1st to 2nd daily dose level** | | | | | | | | |
| --- | --- | --- | --- | --- | --- | --- | --- | --- |
| **Dose 1, md/day** | **Dose 2, md/day** | **Titrated patients** | **Upward titration, %** | | **Downward titration, %** | | **No further titration, %** | |
| **18 (N=1,447)** | -- | -- | 1,447 | 100.0% | 0 | 0.0% | -- | -- |
|  | 27 | 1,139 |  |  |  |  |  |  |
|  | 36 | 26 |  |  |  |  |  |  |
|  | 45 | 274 |  |  |  |  |  |  |
|  | 54 | 3 |  |  |  |  |  |  |
|  | >54 | 5 |  |  |  |  |  |  |
| **27 (N=28)** | 18 | 14 | 14 | 50.0% | 14 | 50.0% | -- | -- |
|  | -- | -- |  |  |  |  |  |  |
|  | 36 | 8 |  |  |  |  |  |  |
|  | 45 | 6 |  |  |  |  |  |  |
|  | 54 | 0 |  |  |  |  |  |  |
|  | >54 | 0 |  |  |  |  |  |  |
| **36 (N=48)** | 18 | 1 | 47 | 97.9% | 1 | 2.1% | -- | -- |
|  | 27 | 0 |  |  |  |  |  |  |
|  | -- | -- |  |  |  |  |  |  |
|  | 45 | 2 |  |  |  |  |  |  |
|  | 54 | 33 |  |  |  |  |  |  |
|  | >54 | 12 |  |  |  |  |  |  |
| **45 (N=91)** | 18 | 28 | 15 | 16.5% | 76 | 83.5% | -- | -- |
|  | 27 | 36 |  |  |  |  |  |  |
|  | 36 | 12 |  |  |  |  |  |  |
|  | -- | -- |  |  |  |  |  |  |
|  | 54 | 10 |  |  |  |  |  |  |
|  | >54 | 5 |  |  |  |  |  |  |
| **54 (N=0)** | 18 | 0 | 0 | 0.0% | 0 | 0.0% | -- | -- |
|  | 27 | 0 |  |  |  |  |  |  |
|  | 36 | 0 |  |  |  |  |  |  |
|  | 45 | 0 |  |  |  |  |  |  |
|  | -- | -- |  |  |  |  |  |  |
|  | >54 | 0 |  |  |  |  |  |  |
| **>54 (N=26)** | 18 | 2 | 0 | 0.0% | 26 | 100.0% | -- | -- |
|  | 27 | 1 |  |  |  |  |  |  |
|  | 36 | 3 |  |  |  |  |  |  |
|  | 45 | 2 |  |  |  |  |  |  |
|  | 54 | 18 |  |  |  |  |  |  |
|  | -- | -- |  |  |  |  |  |  |
| **2nd to 3rd daily dose level** | | | | | | | | |
| **Dose 2, md/day** | **Dose 3, mg/day** | **Titrated patients** | **Upward titration, %** | | **Downward titration, %** | | **No further titration, %** | |
| **18 (N=45)** | -- | -- | 34 | 75.6% | 0 | 0.0% | 11 | 24.4% |
|  | 27 | 12 |  |  |  |  |  |  |
|  | 36 | 4 |  |  |  |  |  |  |
|  | 45 | 16 |  |  |  |  |  |  |
|  | 54 | 0 |  |  |  |  |  |  |
|  | >54 | 2 |  |  |  |  |  |  |
| **27 (N=1,176)** | 18 | 409 | 279 | 23.7% | 409 | 34.8% | 488 | 41.5% |
|  | -- | -- |  |  |  |  |  |  |
|  | 36 | 156 |  |  |  |  |  |  |
|  | 45 | 82 |  |  |  |  |  |  |
|  | 54 | 9 |  |  |  |  |  |  |
|  | >54 | 32 |  |  |  |  |  |  |
| **36 (N=49)** | 18 | 13 | 11 | 22.4% | 19 | 38.8% | 19 | 38.8% |
|  | 27 | 6 |  |  |  |  |  |  |
|  | -- | -- |  |  |  |  |  |  |
|  | 45 | 5 |  |  |  |  |  |  |
|  | 54 | 4 |  |  |  |  |  |  |
|  | >54 | 2 |  |  |  |  |  |  |
| **45 (N=284)** | 18 | 72 | 5 | 1.8% | 151 | 53.2% | 128 | 45.1% |
|  | 27 | 77 |  |  |  |  |  |  |
|  | 36 | 2 |  |  |  |  |  |  |
|  | -- | -- |  |  |  |  |  |  |
|  | 54 | 0 |  |  |  |  |  |  |
|  | >54 | 5 |  |  |  |  |  |  |
| **54 (N=64)** | 18 | 1 | 16 | 25.0% | 35 | 54.7% | 13 | 20.3% |
|  | 27 | 1 |  |  |  |  |  |  |
|  | 36 | 32 |  |  |  |  |  |  |
|  | 45 | 1 |  |  |  |  |  |  |
|  | -- | -- |  |  |  |  |  |  |
|  | >54 | 16 |  |  |  |  |  |  |
| **>54 (N=22)** | 18 | 0 | 0 | 0.0% | 17 | 77.3% | 5 | 22.7% |
|  | 27 | 0 |  |  |  |  |  |  |
|  | 36 | 6 |  |  |  |  |  |  |
|  | 45 | 0 |  |  |  |  |  |  |
|  | 54 | 11 |  |  |  |  |  |  |
|  | -- | -- |  |  |  |  |  |  |
| **3rd to 4th daily dose level** | | | | | | | | |
| **Dose 3, md/day** | **Dose 4, mg/day** | **Titrated patients** | **Upward titration, %** | | **Downward titration, %** | | **No further titration, %** | |
| **18 (N=495)** | -- | -- | 337 | 68.1% | 0 | 0.0% | 158 | 31.9% |
|  | 27 | 191 |  |  |  |  |  |  |
|  | 36 | 23 |  |  |  |  |  |  |
|  | 45 | 120 |  |  |  |  |  |  |
|  | 54 | 2 |  |  |  |  |  |  |
|  | >54 | 1 |  |  |  |  |  |  |
| **27 (N=96)** | 18 | 22 | 39 | 40.6% | 22 | 22.9% | 35 | 36.5% |
|  | -- | -- |  |  |  |  |  |  |
|  | 36 | 7 |  |  |  |  |  |  |
|  | 45 | 24 |  |  |  |  |  |  |
|  | 54 | 2 |  |  |  |  |  |  |
|  | >54 | 6 |  |  |  |  |  |  |
| **36 (N=200)** | 18 | 23 | 71 | 35.5% | 61 | 30.5% | 68 | 34.0% |
|  | 27 | 38 |  |  |  |  |  |  |
|  | -- | -- |  |  |  |  |  |  |
|  | 45 | 33 |  |  |  |  |  |  |
|  | 54 | 13 |  |  |  |  |  |  |
|  | >54 | 25 |  |  |  |  |  |  |
| **45 (N=104)** | 18 | 21 | 7 | 6.7% | 56 | 53.8% | 41 | 39.4% |
|  | 27 | 29 |  |  |  |  |  |  |
|  | 36 | 6 |  |  |  |  |  |  |
|  | -- | -- |  |  |  |  |  |  |
|  | 54 | 1 |  |  |  |  |  |  |
|  | >54 | 6 |  |  |  |  |  |  |
| **54 (N=24)** | 18 | 1 | 5 | 20.8% | 8 | 33.3% | 11 | 45.8% |
|  | 27 | 6 |  |  |  |  |  |  |
|  | 36 | 1 |  |  |  |  |  |  |
|  | 45 | 0 |  |  |  |  |  |  |
|  | -- | -- |  |  |  |  |  |  |
|  | >54 | 5 |  |  |  |  |  |  |
| **>54 (N=57)** | 18 | 1 | 0 | 0.0% | 34 | 59.6% | 23 | 40.4% |
|  | 27 | 12 |  |  |  |  |  |  |
|  | 36 | 13 |  |  |  |  |  |  |
|  | 45 | 1 |  |  |  |  |  |  |
|  | 54 | 7 |  |  |  |  |  |  |
|  | -- | -- |  |  |  |  |  |  |

Child group: 6 to <13 years

**Supplementary Table 4.** Numbers of OROS-MPH users at each dose level among patients with at least one dose titration (Data for Fig. 2d: adolescent group in the Japanese cohort, N= 578)

| **1st to 2nd daily dose level** | | | | | | | | |
| --- | --- | --- | --- | --- | --- | --- | --- | --- |
| **Dose 1, md/day** | **Dose 2, md/day** | **Titrated patients** | **Upward titration, %** | | **Downward titration, %** | | **No further titration, %** | |
| **18 (N=457)** | -- | -- | 457 | 100.0% | 0 | 0.0% | -- | -- |
|  | 27 | 376 |  |  |  |  |  |  |
|  | 36 | 25 |  |  |  |  |  |  |
|  | 45 | 47 |  |  |  |  |  |  |
|  | 54 | 3 |  |  |  |  |  |  |
|  | >54 | 6 |  |  |  |  |  |  |
| **27 (N=35)** | 18 | 18 | 17 | 48.6% | 18 | 51.4% | -- | -- |
|  | -- | -- |  |  |  |  |  |  |
|  | 36 | 9 |  |  |  |  |  |  |
|  | 45 | 3 |  |  |  |  |  |  |
|  | 54 | 2 |  |  |  |  |  |  |
|  | >54 | 3 |  |  |  |  |  |  |
| **36 (N=21)** | 18 | 0 | 21 | 100.0% | 0 | 0.0% | -- | -- |
|  | 27 | 0 |  |  |  |  |  |  |
|  | -- | -- |  |  |  |  |  |  |
|  | 45 | 2 |  |  |  |  |  |  |
|  | 54 | 14 |  |  |  |  |  |  |
|  | >54 | 5 |  |  |  |  |  |  |
| **45 (N=42)** | 18 | 5 | 12 | 28.6% | 30 | 71.4% | -- | -- |
|  | 27 | 14 |  |  |  |  |  |  |
|  | 36 | 11 |  |  |  |  |  |  |
|  | -- | -- |  |  |  |  |  |  |
|  | 54 | 0 |  |  |  |  |  |  |
|  | >54 | 12 |  |  |  |  |  |  |
| **54 (N=3)** | 18 | 0 | 0 | 0.0% | 3 | 100.0% | -- | -- |
|  | 27 | 1 |  |  |  |  |  |  |
|  | 36 | 2 |  |  |  |  |  |  |
|  | 45 | 0 |  |  |  |  |  |  |
|  | -- | -- |  |  |  |  |  |  |
|  | >54 | 0 |  |  |  |  |  |  |
| **>54 (N=20)** | 18 | 3 | 0 | 0.0% | 20 | 100.0% | -- | -- |
|  | 27 | 4 |  |  |  |  |  |  |
|  | 36 | 5 |  |  |  |  |  |  |
|  | 45 | 3 |  |  |  |  |  |  |
|  | 54 | 5 |  |  |  |  |  |  |
|  | -- | -- |  |  |  |  |  |  |
| **2nd to 3rd daily dose level** | | | | | | | | |
| **Dose 2, md/day** | **Dose 3, mg/day** | **Titrated patients** | **Upward titration, %** | | **Downward titration, %** | | **No further titration, %** | |
| **18 (N=26)** | -- | -- | 17 | 65.4% | 0 | 0.0% | 9 | 34.6% |
|  | 27 | 7 |  |  |  |  |  |  |
|  | 36 | 2 |  |  |  |  |  |  |
|  | 45 | 8 |  |  |  |  |  |  |
|  | 54 | 0 |  |  |  |  |  |  |
|  | >54 | 0 |  |  |  |  |  |  |
| **27 (N=395)** | 18 | 105 | 126 | 31.9% | 105 | 26.6% | 164 | 41.5% |
|  | -- | -- |  |  |  |  |  |  |
|  | 36 | 84 |  |  |  |  |  |  |
|  | 45 | 28 |  |  |  |  |  |  |
|  | 54 | 5 |  |  |  |  |  |  |
|  | >54 | 9 |  |  |  |  |  |  |
| **36 (N=52)** | 18 | 6 | 15 | 28.8% | 13 | 25.0% | 24 | 46.2% |
|  | 27 | 7 |  |  |  |  |  |  |
|  | -- | -- |  |  |  |  |  |  |
|  | 45 | 10 |  |  |  |  |  |  |
|  | 54 | 3 |  |  |  |  |  |  |
|  | >54 | 2 |  |  |  |  |  |  |
| **45 (N=55)** | 18 | 8 | 2 | 3.6% | 30 | 54.5% | 23 | 41.8% |
|  | 27 | 19 |  |  |  |  |  |  |
|  | 36 | 3 |  |  |  |  |  |  |
|  | -- | -- |  |  |  |  |  |  |
|  | 54 | 0 |  |  |  |  |  |  |
|  | >54 | 2 |  |  |  |  |  |  |
| **54 (N=24)** | 18 | 0 | 6 | 25.0% | 6 | 25.0% | 12 | 50.0% |
|  | 27 | 1 |  |  |  |  |  |  |
|  | 36 | 5 |  |  |  |  |  |  |
|  | 45 | 0 |  |  |  |  |  |  |
|  | -- | -- |  |  |  |  |  |  |
|  | >54 | 6 |  |  |  |  |  |  |
| **>54 (N=26)** | 18 | 0 | 0 | 0.0% | 16 | 61.5% | 10 | 38.5% |
|  | 27 | 1 |  |  |  |  |  |  |
|  | 36 | 9 |  |  |  |  |  |  |
|  | 45 | 3 |  |  |  |  |  |  |
|  | 54 | 3 |  |  |  |  |  |  |
|  | -- | -- |  |  |  |  |  |  |
| **3rd to 4th daily dose level** | | | | | | | | |
| **Dose 3, md/day** | **Dose 4, mg/day** | **Titrated patients** | **Upward titration, %** | | **Downward titration, %** | | **No further titration, %** | |
| **18 (N=119)** | -- | -- | 80 | 67.2% | 0 | 0.0% | 39 | 32.8% |
|  | 27 | 43 |  |  |  |  |  |  |
|  | 36 | 5 |  |  |  |  |  |  |
|  | 45 | 31 |  |  |  |  |  |  |
|  | 54 | 0 |  |  |  |  |  |  |
|  | >54 | 1 |  |  |  |  |  |  |
| **27 (N=35)** | 18 | 12 | 10 | 28.6% | 12 | 34.3% | 13 | 37.1% |
|  | -- | -- |  |  |  |  |  |  |
|  | 36 | 3 |  |  |  |  |  |  |
|  | 45 | 6 |  |  |  |  |  |  |
|  | 54 | 0 |  |  |  |  |  |  |
|  | >54 | 1 |  |  |  |  |  |  |
| **36 (N=103)** | 18 | 7 | 33 | 32.0% | 26 | 25.2% | 44 | 42.7% |
|  | 27 | 19 |  |  |  |  |  |  |
|  | -- | -- |  |  |  |  |  |  |
|  | 45 | 27 |  |  |  |  |  |  |
|  | 54 | 3 |  |  |  |  |  |  |
|  | >54 | 3 |  |  |  |  |  |  |
| **45 (N=49)** | 18 | 4 | 3 | 6.1% | 25 | 51.0% | 21 | 42.9% |
|  | 27 | 17 |  |  |  |  |  |  |
|  | 36 | 4 |  |  |  |  |  |  |
|  | -- | -- |  |  |  |  |  |  |
|  | 54 | 0 |  |  |  |  |  |  |
|  | >54 | 3 |  |  |  |  |  |  |
| **54 (N=11)** | 18 | 1 | 2 | 18.2% | 7 | 63.6% | 2 | 18.2% |
|  | 27 | 4 |  |  |  |  |  |  |
|  | 36 | 1 |  |  |  |  |  |  |
|  | 45 | 1 |  |  |  |  |  |  |
|  | -- | -- |  |  |  |  |  |  |
|  | >54 | 2 |  |  |  |  |  |  |
| **>54 (N=19)** | 18 | 0 | 0 | 0.0% | 8 | 42.1% | 11 | 57.9% |
|  | 27 | 1 |  |  |  |  |  |  |
|  | 36 | 5 |  |  |  |  |  |  |
|  | 45 | 1 |  |  |  |  |  |  |
|  | 54 | 1 |  |  |  |  |  |  |
|  | -- | -- |  |  |  |  |  |  |

Adolescent group: 13 to <18 years

**Supplementary Table 5.** Numbers of OROS-MPH users at each dose level among MPH-naïve patients with at least one dose titration (Data for Supplementary Fig 1a: child group of sensitivity analysis in the US cohort, N=23,901)

| **1st to 2nd daily dose level** | | | | | | | | |
| --- | --- | --- | --- | --- | --- | --- | --- | --- |
| **Dose 1, md/day** | **Dose 2, md/day** | **Titrated patients** | **Upward titration, %** | | **Downward titration, %** | | **No further titration, %** | |
| **18 (N=14,854)** | -- | -- | 14,854 | 100.0% | 0 | 0.0% | -- | -- |
|  | 27 | 9,446 |  |  |  |  |  |  |
|  | 36 | 4,615 |  |  |  |  |  |  |
|  | 45 | 93 |  |  |  |  |  |  |
|  | 54 | 656 |  |  |  |  |  |  |
|  | >54 | 44 |  |  |  |  |  |  |
| **27 (N=3,382)** | 18 | 512 | 2,870 | 84.9% | 512 | 15.1% | -- | -- |
|  | -- | -- |  |  |  |  |  |  |
|  | 36 | 2,668 |  |  |  |  |  |  |
|  | 45 | 43 |  |  |  |  |  |  |
|  | 54 | 120 |  |  |  |  |  |  |
|  | >54 | 39 |  |  |  |  |  |  |
| **36 (N=3,426)** | 18 | 654 | 2,269 | 66.2% | 1,157 | 33.8% | -- | -- |
|  | 27 | 503 |  |  |  |  |  |  |
|  | -- | -- |  |  |  |  |  |  |
|  | 45 | 196 |  |  |  |  |  |  |
|  | 54 | 1,853 |  |  |  |  |  |  |
|  | >54 | 220 |  |  |  |  |  |  |
| **45 (N=446)** | 18 | 54 | 230 | 51.6% | 216 | 48.4% | -- | -- |
|  | 27 | 93 |  |  |  |  |  |  |
|  | 36 | 69 |  |  |  |  |  |  |
|  | -- | -- |  |  |  |  |  |  |
|  | 54 | 159 |  |  |  |  |  |  |
|  | >54 | 71 |  |  |  |  |  |  |
| **54 (N=1,465)** | 18 | 251 | 394 | 26.9% | 1,071 | 73.1% | -- | -- |
|  | 27 | 60 |  |  |  |  |  |  |
|  | 36 | 747 |  |  |  |  |  |  |
|  | 45 | 13 |  |  |  |  |  |  |
|  | -- | -- |  |  |  |  |  |  |
|  | >54 | 394 |  |  |  |  |  |  |
| **>54 (N=328)** | 18 | 10 | 0 | 0.0% | 328 | 100.0% | -- | -- |
|  | 27 | 16 |  |  |  |  |  |  |
|  | 36 | 123 |  |  |  |  |  |  |
|  | 45 | 2 |  |  |  |  |  |  |
|  | 54 | 177 |  |  |  |  |  |  |
|  | -- | -- |  |  |  |  |  |  |
| **2nd to 3rd daily dose level** | | | | | | | | |
| **Dose 2, md/day** | **Dose 3, mg/day** | **Titrated patients** | **Upward titration, %** | | **Downward titration, %** | | **No further titration, %** | |
| **18 (N=1,481)** | -- | -- | 885 | 59.8% | 0 | 0.0% | 596 | 40.2% |
|  | 27 | 373 |  |  |  |  |  |  |
|  | 36 | 260 |  |  |  |  |  |  |
|  | 45 | 13 |  |  |  |  |  |  |
|  | 54 | 215 |  |  |  |  |  |  |
|  | >54 | 24 |  |  |  |  |  |  |
| **27 (N=10,118)** | 18 | 1,284 | 3,983 | 39.4% | 1,284 | 12.7% | 4,851 | 47.9% |
|  | -- | -- |  |  |  |  |  |  |
|  | 36 | 3,726 |  |  |  |  |  |  |
|  | 45 | 100 |  |  |  |  |  |  |
|  | 54 | 118 |  |  |  |  |  |  |
|  | >54 | 39 |  |  |  |  |  |  |
| **36 (N=8,222)** | 18 | 1,076 | 2,410 | 29.3% | 1,980 | 24.1% | 3,832 | 46.6% |
|  | 27 | 904 |  |  |  |  |  |  |
|  | -- | -- |  |  |  |  |  |  |
|  | 45 | 254 |  |  |  |  |  |  |
|  | 54 | 2,004 |  |  |  |  |  |  |
|  | >54 | 152 |  |  |  |  |  |  |
| **45 (N=347)** | 18 | 35 | 72 | 20.7% | 136 | 39.2% | 139 | 40.1% |
|  | 27 | 67 |  |  |  |  |  |  |
|  | 36 | 34 |  |  |  |  |  |  |
|  | -- | -- |  |  |  |  |  |  |
|  | 54 | 69 |  |  |  |  |  |  |
|  | >54 | 3 |  |  |  |  |  |  |
| **54 (N=2,965)** | 18 | 248 | 406 | 13.7% | 1,329 | 44.8% | 1,230 | 41.5% |
|  | 27 | 89 |  |  |  |  |  |  |
|  | 36 | 967 |  |  |  |  |  |  |
|  | 45 | 25 |  |  |  |  |  |  |
|  | -- | -- |  |  |  |  |  |  |
|  | >54 | 406 |  |  |  |  |  |  |
| **>54 (N=768)** | 18 | 18 | 0 | 0.0% | 436 | 56.8% | 332 | 43.2% |
|  | 27 | 26 |  |  |  |  |  |  |
|  | 36 | 217 |  |  |  |  |  |  |
|  | 45 | 3 |  |  |  |  |  |  |
|  | 54 | 172 |  |  |  |  |  |  |
|  | -- | -- |  |  |  |  |  |  |
| **3rd to 4th daily dose level** | | | | | | | | |
| **Dose 3, md/day** | **Dose 4, mg/day** | **Titrated patients** | **Upward titration, %** | | **Downward titration, %** | | **No further titration, %** | |
| **18 (N=2,661)** | -- | -- | 1,553 | 58.4% | 0 | 0.0% | 1,108 | 41.6% |
|  | 27 | 752 |  |  |  |  |  |  |
|  | 36 | 467 |  |  |  |  |  |  |
|  | 45 | 52 |  |  |  |  |  |  |
|  | 54 | 257 |  |  |  |  |  |  |
|  | >54 | 25 |  |  |  |  |  |  |
| **27 (N=1,459)** | 18 | 164 | 655 | 44.9% | 164 | 11.2% | 640 | 43.9% |
|  | -- | -- |  |  |  |  |  |  |
|  | 36 | 543 |  |  |  |  |  |  |
|  | 45 | 40 |  |  |  |  |  |  |
|  | 54 | 47 |  |  |  |  |  |  |
|  | >54 | 25 |  |  |  |  |  |  |
| **36 (N=5,204)** | 18 | 392 | 1,645 | 31.6% | 1,139 | 21.9% | 2,420 | 46.5% |
|  | 27 | 747 |  |  |  |  |  |  |
|  | -- | -- |  |  |  |  |  |  |
|  | 45 | 227 |  |  |  |  |  |  |
|  | 54 | 1,249 |  |  |  |  |  |  |
|  | >54 | 169 |  |  |  |  |  |  |
| **45 (N=395)** | 18 | 34 | 83 | 21.0% | 158 | 40.0% | 154 | 39.0% |
|  | 27 | 76 |  |  |  |  |  |  |
|  | 36 | 48 |  |  |  |  |  |  |
|  | -- | -- |  |  |  |  |  |  |
|  | 54 | 80 |  |  |  |  |  |  |
|  | >54 | 3 |  |  |  |  |  |  |
| **54 (N=2,578)** | 18 | 251 | 224 | 8.7% | 1,212 | 47.0% | 1,142 | 44.3% |
|  | 27 | 93 |  |  |  |  |  |  |
|  | 36 | 827 |  |  |  |  |  |  |
|  | 45 | 41 |  |  |  |  |  |  |
|  | -- | -- |  |  |  |  |  |  |
|  | >54 | 224 |  |  |  |  |  |  |
| **>54 (N=624)** | 18 | 11 | 0 | 0.0% | 335 | 53.7% | 289 | 46.3% |
|  | 27 | 21 |  |  |  |  |  |  |
|  | 36 | 125 |  |  |  |  |  |  |
|  | 45 | 2 |  |  |  |  |  |  |
|  | 54 | 176 |  |  |  |  |  |  |
|  | -- | -- |  |  |  |  |  |  |

Child group: 6 to <13 years

**Supplementary Table 6.** Numbers of OROS-MPH users at each dose level among MPH-naïve patients with at least one dose titration (Data for Supplementary Fig 1b: adolescent group of sensitivity analysis in the US cohort, N=9,512)

| **1st to 2nd daily dose level** | | | | | | | | |
| --- | --- | --- | --- | --- | --- | --- | --- | --- |
| **Dose 1, md/day** | **Dose 2, md/day** | **Titrated patients** | **Upward titration, %** | | **Downward titration, %** | | **No further titration, %** | |
| **18 (N=4,294)** | -- | -- | 4,294 | 100.0% | 0 | 0.0% | -- | -- |
|  | 27 | 1,854 |  |  |  |  |  |  |
|  | 36 | 1,818 |  |  |  |  |  |  |
|  | 45 | 30 |  |  |  |  |  |  |
|  | 54 | 565 |  |  |  |  |  |  |
|  | >54 | 27 |  |  |  |  |  |  |
| **27 (N=1,354)** | 18 | 163 | 1,191 | 88.0% | 163 | 12.0% | -- | -- |
|  | -- | -- |  |  |  |  |  |  |
|  | 36 | 1,048 |  |  |  |  |  |  |
|  | 45 | 14 |  |  |  |  |  |  |
|  | 54 | 111 |  |  |  |  |  |  |
|  | >54 | 18 |  |  |  |  |  |  |
| **36 (N=2,222)** | 18 | 383 | 1,608 | 72.4% | 614 | 27.6% | -- | -- |
|  | 27 | 231 |  |  |  |  |  |  |
|  | -- | -- |  |  |  |  |  |  |
|  | 45 | 70 |  |  |  |  |  |  |
|  | 54 | 1,411 |  |  |  |  |  |  |
|  | >54 | 127 |  |  |  |  |  |  |
| **45 (N=190)** | 18 | 20 | 84 | 44.2% | 106 | 55.8% | -- | -- |
|  | 27 | 56 |  |  |  |  |  |  |
|  | 36 | 30 |  |  |  |  |  |  |
|  | -- | -- |  |  |  |  |  |  |
|  | 54 | 33 |  |  |  |  |  |  |
|  | >54 | 51 |  |  |  |  |  |  |
| **54 (N=1,203)** | 18 | 260 | 229 | 19.0% | 974 | 81.0% | -- | -- |
|  | 27 | 61 |  |  |  |  |  |  |
|  | 36 | 636 |  |  |  |  |  |  |
|  | 45 | 17 |  |  |  |  |  |  |
|  | -- | -- |  |  |  |  |  |  |
|  | >54 | 229 |  |  |  |  |  |  |
| **>54 (N=249)** | 18 | 21 | 0 | 0.0% | 249 | 100.0% | -- | -- |
|  | 27 | 14 |  |  |  |  |  |  |
|  | 36 | 94 |  |  |  |  |  |  |
|  | 45 | 6 |  |  |  |  |  |  |
|  | 54 | 114 |  |  |  |  |  |  |
|  | -- | -- |  |  |  |  |  |  |
| **2nd to 3rd daily dose level** | | | | | | | | |
| **Dose 2, md/day** | **Dose 3, mg/day** | **Titrated patients** | **Upward titration, %** | | **Downward titration, %** | | **No further titration, %** | |
| **18 (N=847)** | -- | -- | 444 | 52.4% | 0 | 0.0% | 403 | 47.6% |
|  | 27 | 92 |  |  |  |  |  |  |
|  | 36 | 155 |  |  |  |  |  |  |
|  | 45 | 6 |  |  |  |  |  |  |
|  | 54 | 157 |  |  |  |  |  |  |
|  | >54 | 34 |  |  |  |  |  |  |
| **27 (N=2,216)** | 18 | 222 | 787 | 35.5% | 222 | 10.0% | 1,207 | 54.5% |
|  | -- | -- |  |  |  |  |  |  |
|  | 36 | 691 |  |  |  |  |  |  |
|  | 45 | 16 |  |  |  |  |  |  |
|  | 54 | 66 |  |  |  |  |  |  |
|  | >54 | 14 |  |  |  |  |  |  |
| **36 (N=3,626)** | 18 | 374 | 1,077 | 29.7% | 579 | 16.0% | 1,970 | 54.3% |
|  | 27 | 205 |  |  |  |  |  |  |
|  | -- | -- |  |  |  |  |  |  |
|  | 45 | 55 |  |  |  |  |  |  |
|  | 54 | 934 |  |  |  |  |  |  |
|  | >54 | 88 |  |  |  |  |  |  |
| **45 (N=137)** | 18 | 20 | 24 | 17.5% | 44 | 32.1% | 69 | 50.4% |
|  | 27 | 12 |  |  |  |  |  |  |
|  | 36 | 12 |  |  |  |  |  |  |
|  | -- | -- |  |  |  |  |  |  |
|  | 54 | 21 |  |  |  |  |  |  |
|  | >54 | 3 |  |  |  |  |  |  |
| **54 (N=2,234)** | 18 | 228 | 154 | 6.9% | 873 | 39.1% | 1,207 | 54.0% |
|  | 27 | 51 |  |  |  |  |  |  |
|  | 36 | 579 |  |  |  |  |  |  |
|  | 45 | 15 |  |  |  |  |  |  |
|  | -- | -- |  |  |  |  |  |  |
|  | >54 | 154 |  |  |  |  |  |  |
| **>54 (N=452)** | 18 | 19 | 0 | 0.0% | 205 | 45.4% | 247 | 54.6% |
|  | 27 | 11 |  |  |  |  |  |  |
|  | 36 | 61 |  |  |  |  |  |  |
|  | 45 | 0 |  |  |  |  |  |  |
|  | 54 | 114 |  |  |  |  |  |  |
|  | -- | -- |  |  |  |  |  |  |
| **3rd to 4th daily dose level** | | | | | | | | |
| **Dose 3, md/day** | **Dose 4, mg/day** | **Titrated patients** | **Upward titration, %** | | **Downward titration, %** | | **No further titration, %** | |
| **18 (N=863)** | -- | -- | 421 | 48.8% | 0 | 0.0% | 442 | 51.2% |
|  | 27 | 111 |  |  |  |  |  |  |
|  | 36 | 140 |  |  |  |  |  |  |
|  | 45 | 21 |  |  |  |  |  |  |
|  | 54 | 120 |  |  |  |  |  |  |
|  | >54 | 29 |  |  |  |  |  |  |
| **27 (N=371)** | 18 | 34 | 127 | 34.2% | 34 | 9.2% | 210 | 56.6% |
|  | -- | -- |  |  |  |  |  |  |
|  | 36 | 87 |  |  |  |  |  |  |
|  | 45 | 9 |  |  |  |  |  |  |
|  | 54 | 22 |  |  |  |  |  |  |
|  | >54 | 9 |  |  |  |  |  |  |
| **36 (N=1,498)** | 18 | 125 | 468 | 31.2% | 239 | 16.0% | 791 | 52.8% |
|  | 27 | 114 |  |  |  |  |  |  |
|  | -- | -- |  |  |  |  |  |  |
|  | 45 | 40 |  |  |  |  |  |  |
|  | 54 | 374 |  |  |  |  |  |  |
|  | >54 | 54 |  |  |  |  |  |  |
| **45 (N=92)** | 18 | 11 | 9 | 9.8% | 36 | 39.1% | 47 | 51.1% |
|  | 27 | 14 |  |  |  |  |  |  |
|  | 36 | 11 |  |  |  |  |  |  |
|  | -- | -- |  |  |  |  |  |  |
|  | 54 | 8 |  |  |  |  |  |  |
|  | >54 | 1 |  |  |  |  |  |  |
| **54 (N=1,292)** | 18 | 151 | 122 | 9.4% | 539 | 41.7% | 631 | 48.8% |
|  | 27 | 47 |  |  |  |  |  |  |
|  | 36 | 330 |  |  |  |  |  |  |
|  | 45 | 11 |  |  |  |  |  |  |
|  | -- | -- |  |  |  |  |  |  |
|  | >54 | 122 |  |  |  |  |  |  |
| **>54 (N=293)** | 18 | 11 | 0 | 0.0% | 156 | 53.2% | 137 | 46.8% |
|  | 27 | 9 |  |  |  |  |  |  |
|  | 36 | 62 |  |  |  |  |  |  |
|  | 45 | 1 |  |  |  |  |  |  |
|  | 54 | 73 |  |  |  |  |  |  |
|  | -- | -- |  |  |  |  |  |  |

Adolescent group: 13 to <18 years;

**Supplementary Fig. 1**

Sensitivity analysis: Sankey diagrams of OROS-MPH dose titration patterns among MPH-naïve ADHD patients with at least one titration: Supplementary Fig. 1a Child OROS-MPH users in the US cohort (N=23,901); Supplementary Fig. 1b Adolescent OROS-MPH users in the US cohort (N=9,512). ‘1_18’ refers to patients whose initial OROS-MPH daily dose level was 18 mg/day, ‘2_27’ refers to patients whose second OROS-MPH daily dose level was 27 mg/day, and so on.


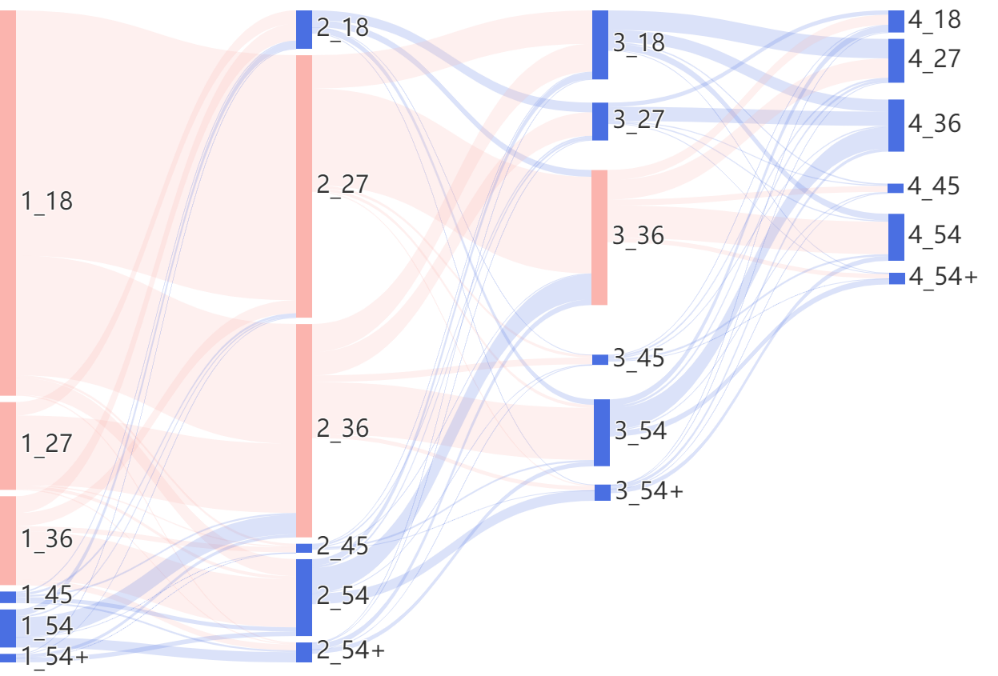


Supplementary Fig. 1a


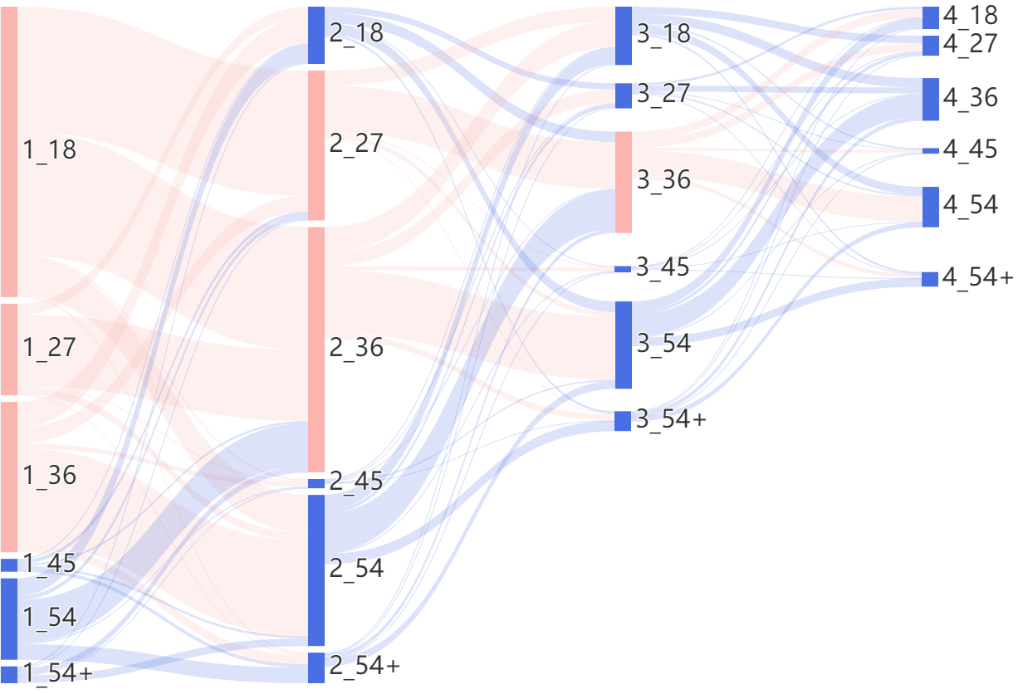


Supplementary Fig. 1b
